# Supplementary material for: BACH1 controls hepatic insulin signaling and glucose homeostasis in mice
Source: Nat Commun. 2023 Dec 21;14:8428. doi: 10.1038/s41467-023-44088-z (PMC10739811; doi:10.1038/s41467-023-44088-z)

BACH1 Controls Hepatic Insulin Signaling and Glucose Homeostasis in Mice

SUPPLEMENTARY INFORMATION

This PDF file includes:

- Supplemental Tables S1-S6
- Supplemental Figures S1-S13
- Uncropped Scans of Blots in Supplementary Figures

Supplementary Tables

Table S1. Sequences for shRNA.

Gene Name	Sequence
Ptpn1-shRNA（5'-3'）	CCAGGACAUUCGACAUGAATT
Bach1-shRNA（5'-3'）	GCGUACACAAUAUCGAGGATT

The sequences of the short hairpin RNA (shRNA) targeting mouse *Ptpn1* or *Bach1*.

Table S2. Primers for genotyping the mice.

Primer	Sequence
floxed *Bach1*-1 forward (5'-3')	TGTCTGTCTGTATGATGGGCATG
floxed *Bach1*-1 reverse (5'-3')	AAGGGTTATTGAATATGATCGGA
floxed *Bach1*-2 forward (5'-3')	GCATCGCATTGTCTGAGTAGGTG
floxed *Bach1-2* reverse (5'-3')	CATCTTTCTGTACCGTTCCCCTA

The sequences of the primers used for the PCR genotyping of *Bach1*$^{flox/flox}$ mice.

Table S3. Information of the human liver samples.

Symptom		Gender	Age	Steatosis
Normal	Normal-1	Male	Median age 41 (range 28-67)	No
	Normal-2	Male		No
	Normal-3	Male		No
	Normal-4	Female		No
	Normal-5	Female		No
	Normal-6	Female		No
NAFLD	NAFLD-1	Male	Median age 36 (range 26-54)	40% steatosis
	NAFLD-2	Male		40% steatosis
	NAFLD-3	Male		10% steatosis
	NAFLD-4	Female		50% steatosis
	NAFLD-5	Female		20% steatosis
	NAFLD-6	Female		15% steatosis

The information including age, gender, and the degree of steatosis in the human liver samples is shown. Human liver samples were obtained from adult patients with non-alcoholic fatty liver disease (NAFLD) (n=6, median age 36) or donors whose livers were excluded from liver transplantation for non-hepatic reasons (Normal, n=6, median age 41).

Table S4. Primers for qRT-PCR.

Primer	Sequence (5’-3’)
β-Actin-F (mouse)	GGCTGTATTCCCCTCCATCG
β-Actin-R (mouse)	CCAGTTGGTAACAATGCCATGT
Bach1-F (mouse)	TGTGATTAGCCTGGGAGA
Bach1-R (mouse)	CGATTTCCGACTCAAGGT
Pck1-F (mouse)	CTGCATAACGGTCTGGACTT
Pck1-R (mouse)	CAGCAACTGCCCGTACTCC
G6pc-F (mouse)	CGACTCGCTATCTCCAAGTGA
G6pc -R (mouse)	GGGCGTTGTCCAAACAGAAT
BACH1-F (human)	CGCCTCAGCTCTGGTTGATG
BACH1-R (human)	CATCAGCCTGGCCTACGATT
β-ACTIN-F (human)	CATGTACGTTGCTATCCAGGC
β-ACTIN-R (human)	CTCCTTAATGTCACGCACGAT

The sequences of the primers used for detection of human *BACH1*, *β-ACTIN* and mouse *Bach1*, *β-Actin*, *Pck1*, *G6pc* by real-time reverse transcription-PCR (qRT-PCR).

Table S5. Sequences of gene-siRNA.

Gene Name	Sequence
Scramble	UUCUCCGAACGUGUCACGUTT
BACH1-siRNA1 (5'-3')	CCGCAGGUAUCAAGG AAAUTT
BACH1-siRNA2 (5'-3')	GUCAGGAUUUACCUUUGAATT
BACH1-siRNA3 (5'-3')	CCAGGUCAAAGGACUUUCATT
Ptpn1-siRNA1 (5'-3')	CCAGGAAGAUAAUGACUAUTT
Ptpn1-siRNA2 (5'-3')	CCGUGGACAUCAAGAAAGUTT
Ptpn1-siRNA3 (5'-3')	CCAGGACAUUCGACAUGAATT

The sequences of the siRNAs targeting human *BACH1* or mouse *Ptpn1*.

Table S6. Antibodies for immunoblotting and immunofluorescence.

Target antigen	Vendor or Source	Catalog #	Working concentration
BACH1	Santa Cruz	sc-271211	WB 1:200
			IP 3 μg
BACH1	Proteintech	14018-1-AP	IHC 1:200
			IF 1:200
β-ACTIN	Proteintech	66009-1-lg	WB 1:10000
IR-β	Cell Signaling Technology	3025	WB 1:1000
FLAG	Sigma-Aldrich	F1804	WB 1:1000
HA	Santa Cruz	sc-7392	WB 1:200
p-IR-β	Cell Signaling Technology	3021	WB 1:1000
p-AKT	Cell Signaling Technology	4060	WB 1:1000
AKT	Cell Signaling Technology	4691	WB 1:1000
p-GSK-3β	Cell Signaling Technology	9322	WB 1:1000
GSK-3β	Cell Signaling Technology	12456	WB 1:1000
p-FOXO1	Cell Signaling Technology	2599	WB 1:1000
FOXO1	Cell Signaling Technology	2880	WB 1:1000
PTP1B	Proteintech	11334-1-AP	WB 1:1000
PP2A-Aα	Santa Cruz	sc-56954	WB 1:200
PTEN	Santa Cruz	sc-7974	WB 1:200
LC3B	Cell Signaling Technology	3868	WB 1:1000
			IF 1:200
SQSTM1/p62	Cell Signaling Technology	23214	WB 1:1000
LAMP1	Cell Signaling Technology	9091	WB 1:1000
PLIN2	Abcam	ab108323	WB 1:1000
GFP	Proteintech	50430-2-AP	WB 1:1000
			IF 1:200
ERp57/ERp60 Polyclonal antibody	Proteintech	15967-1-AP	IF 1:200
Alexa-Fluor 488 Donkey Anti-Rabbit lgG	Jackson	711-545-152	IF 1:200
Alexa-Fluor 594 Donkey Anti-Rabbit lgG	Jackson	711-585-152	IF 1:200
Alexa-Fluor 488 Donkey Anti-Mouse lgG	Jackson	715-545-150	IF 1:200
Alexa-Fluor 647 Donkey Anti-Rabbit lgG	Abcam	ab150075	IF 1:200
HRP-Goat Anti-Mouse lgG	Thermo Fisher Scientific	31460	WB 1:5000
			IHC 1:500
HRP-Goat Anti-Rabbit lgG	Thermo Fisher Scientific	31430	WB 1:5000
			IHC 1:500

The information for primary antibodies and secondary antibodies used in the immunoblotting and immunofluorescence.

Supplementary Figures and Figure Legends.

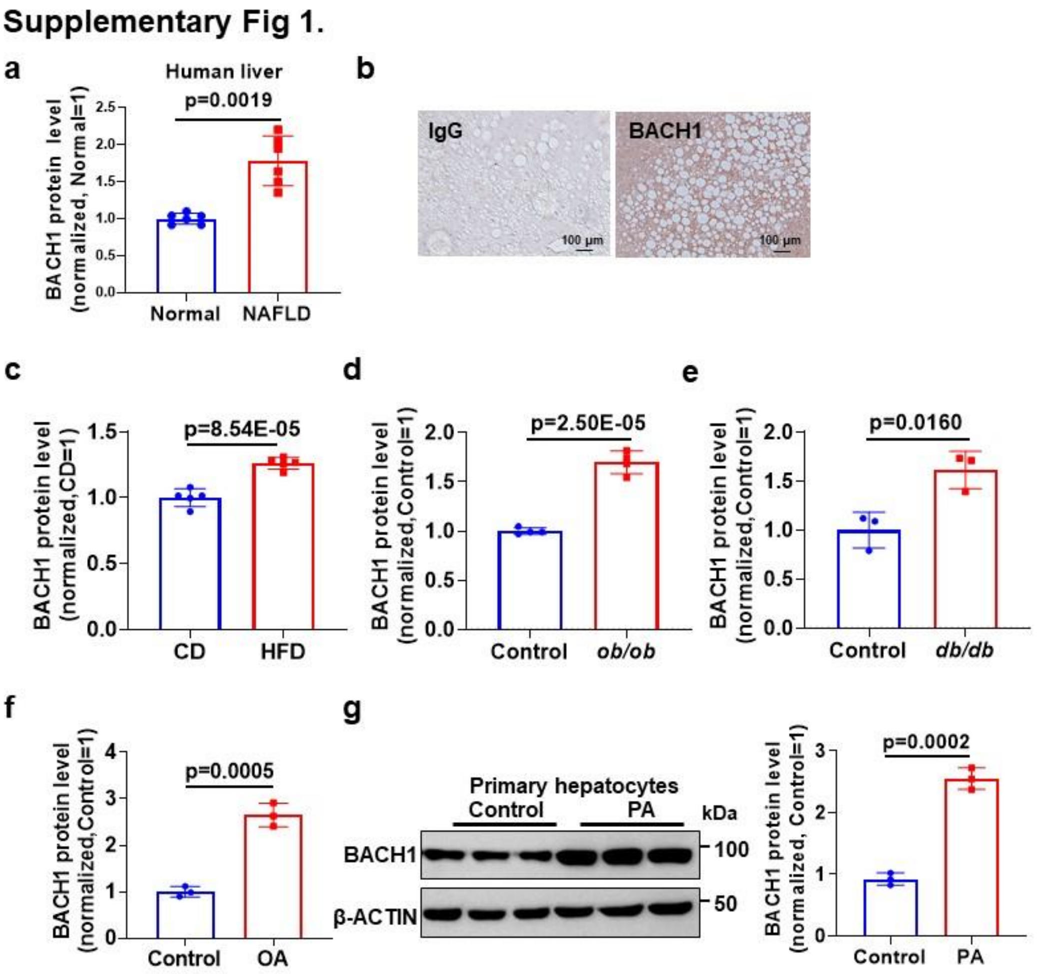

Fig. S1. BACH1 is elevated in the livers of patients with NAFLD and obese mice.

(a) The quantification data of BACH1 protein levels for Fig 1c was shown (n = 6 individuals per group). **(b)** Immunohistochemical staining of BACH1 in livers of NAFLD patients. Rabbit IgG (10 μg/mL) was used as a negative control (n = 6 individuals per group). Scale bar=100 μm. **(c)** The quantification data of BACH1 protein levels for Fig 1h was shown (n=5 mice per group). **(d)** The quantification data of BACH1 protein levels for Fig 1i was shown (n=4 mice per group). **(e)** The quantification data of BACH1 protein levels for Fig 1j was shown (n=3 mice per group). **(f)** The quantification data of BACH1 protein levels for Fig 1k was shown (n=3 mice per group). **(g)** Left: Primary hepatocytes were treated with 1 mM palmitic acid (PA) for 12 hours and then subjected to immunoblot analyses to determine the BACH1 expression (n = 3 biological replicates). Right: The quantification data of BACH1 protein levels for Fig. S1g was shown. Statistical analysis was performed by unpaired two-tailed Student's t-test for a, c, d, e, f, g. Data are presented as mean values ± SD. Source data are provided as a Source Data file.

Supplementary Fig 2.

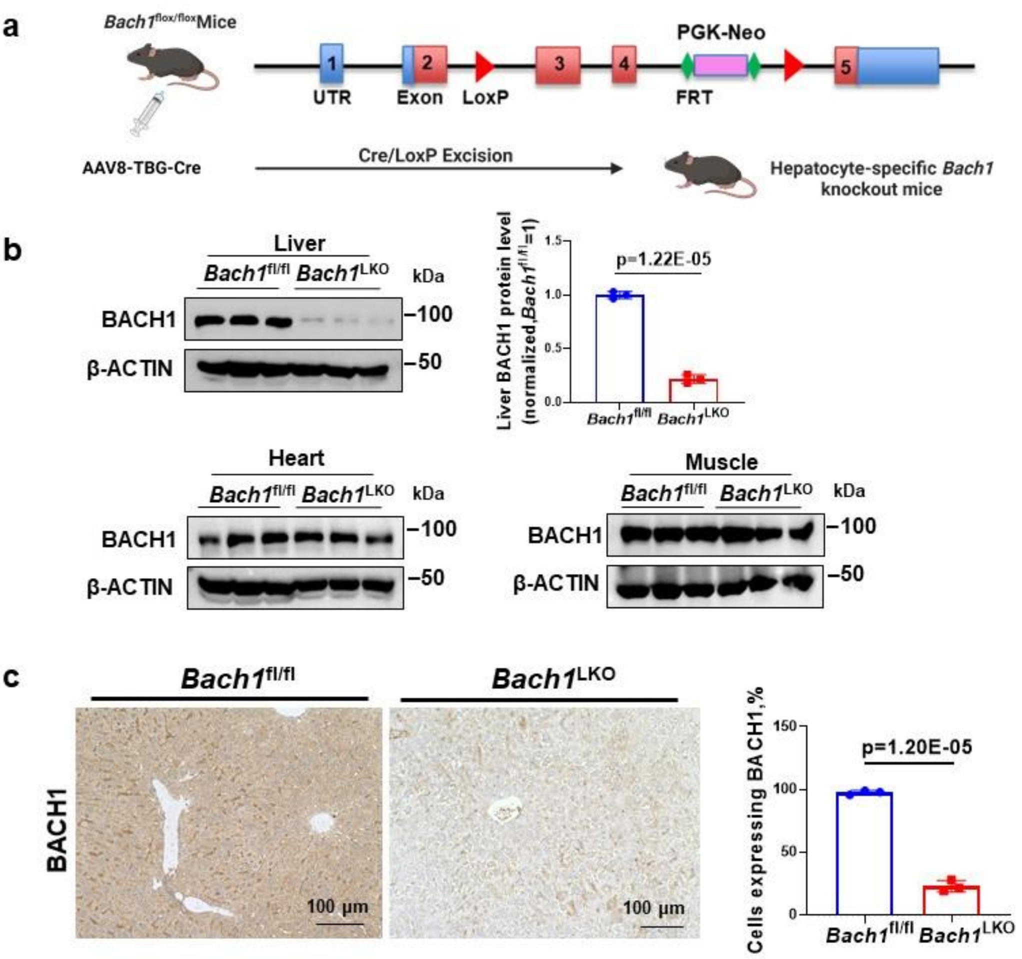

Fig. S2. BACH1 is specifically knocked down in the mouse livers

(a) Schematic diagram depicting the generation of hepatocyte-deficient *Bach1* (*Bach1*^{LKO}) mice. *Bach1*$^{flox/flox}$ mice were injected with AAV8-TBG-Cre. Fig. S2a was created with Biorender under paid subscription (Agreement number: QL26232TDS). **(b)** Representative Western blotting showing the expression of BACH1 in the heart, muscle, and liver samples from *Bach1*$^{fl/fl}$ and *Bach1*^{LKO} mice. The columns showed the quantification data of BACH1 protein levels of the mouse livers (n=3 mice per group). **(c)** Left: The representative images of immunohistochemistry assay of BACH1 in *Bach1*$^{fl/fl}$ and *Bach1*^{LKO} mouse liver samples. Right: Quantitative data of immunohistochemistry assay of BACH1 in the liver tissues (n=3 mice per group). Scale bar=100 μm. Statistical analysis was performed by unpaired two-tailed Student's t-test for b and c. Data are presented as mean values ± SD. Source data are provided as a Source Data file.

Supplementary Fig 3.

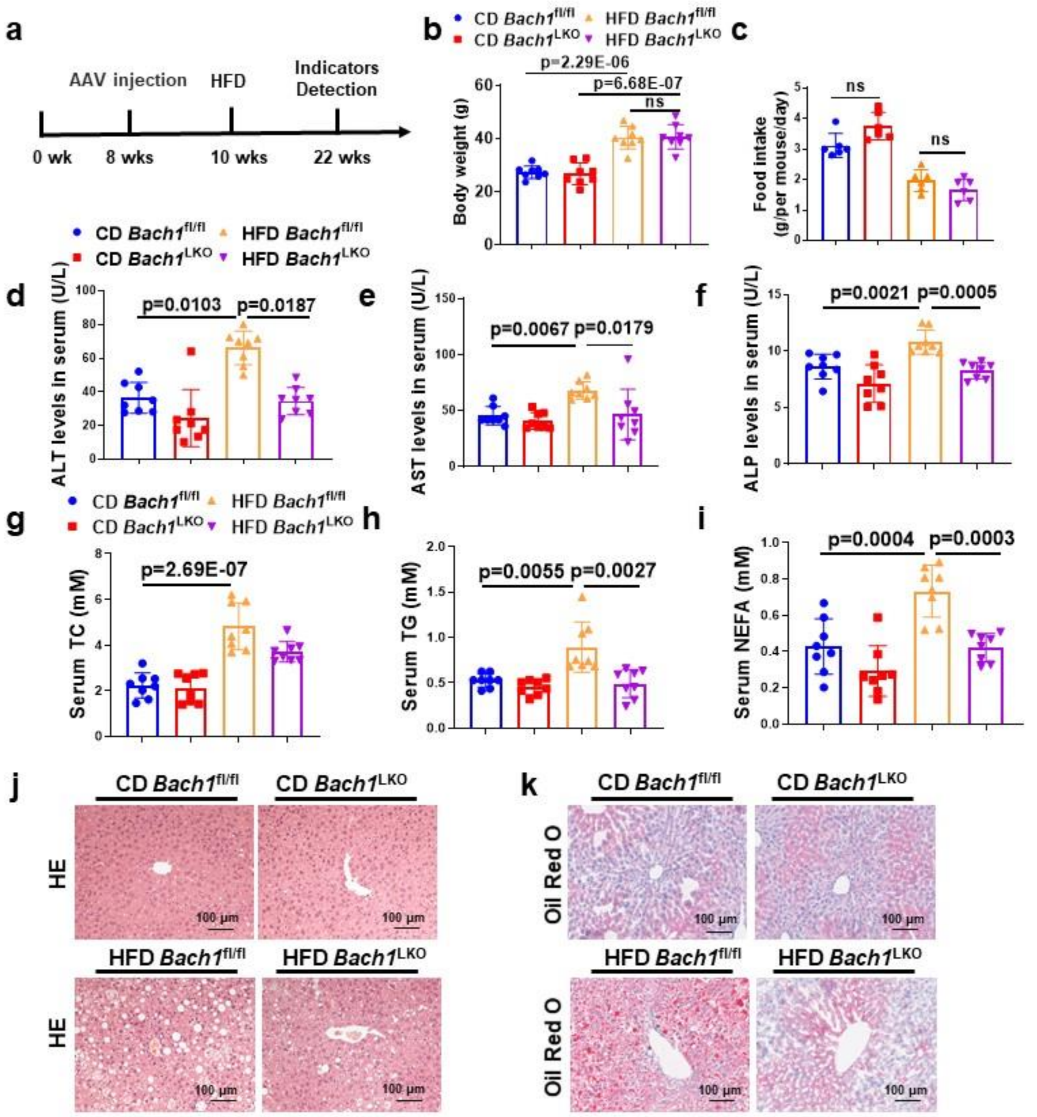

Fig. S3. BACH1 deletion relieves HFD-triggered hepatic steatosis.

(a) Timeline of mouse modeling. **(b)** Body weight (n=8 mice per group) and **(c)** Food intake (n=6 mice per group). **(d-f)** Liver function manifested by serum ALT**(d)**, AST**(e)**, and ALP levels **(f)** of *Bach1*^{LKO} and *Bach1*$^{fl/fl}$ mice fed a CD or HFD for 12 weeks. **(g-i)** TC **(g)**, TG **(h)**, and NEFA **(i)** levels were detected in the serum of *Bach1*$^{fl/fl}$ and *Bach1*^{LKO} mice fed for 12 weeks with a CD or HFD (n=8 mice per group). **(j)** H&E and **(k)** Oil red O staining of liver tissues from *Bach1*$^{fl/fl}$ and *Bach1*^{LKO} mice fed for 12 weeks with CD or HFD (n=8 mice per group). Scale bar=100 μm. Statistical analysis was performed by two-way ANOVA followed by Tukey post hoc tests for b and i, by two-way ANOVA followed by Kruskal-Wallis test with Dunn multiple comparisons test for c, d, e, f, g, and h. Data are presented as mean values ± SD. Source data are provided as a Source Data file.

Supplementary Fig 4

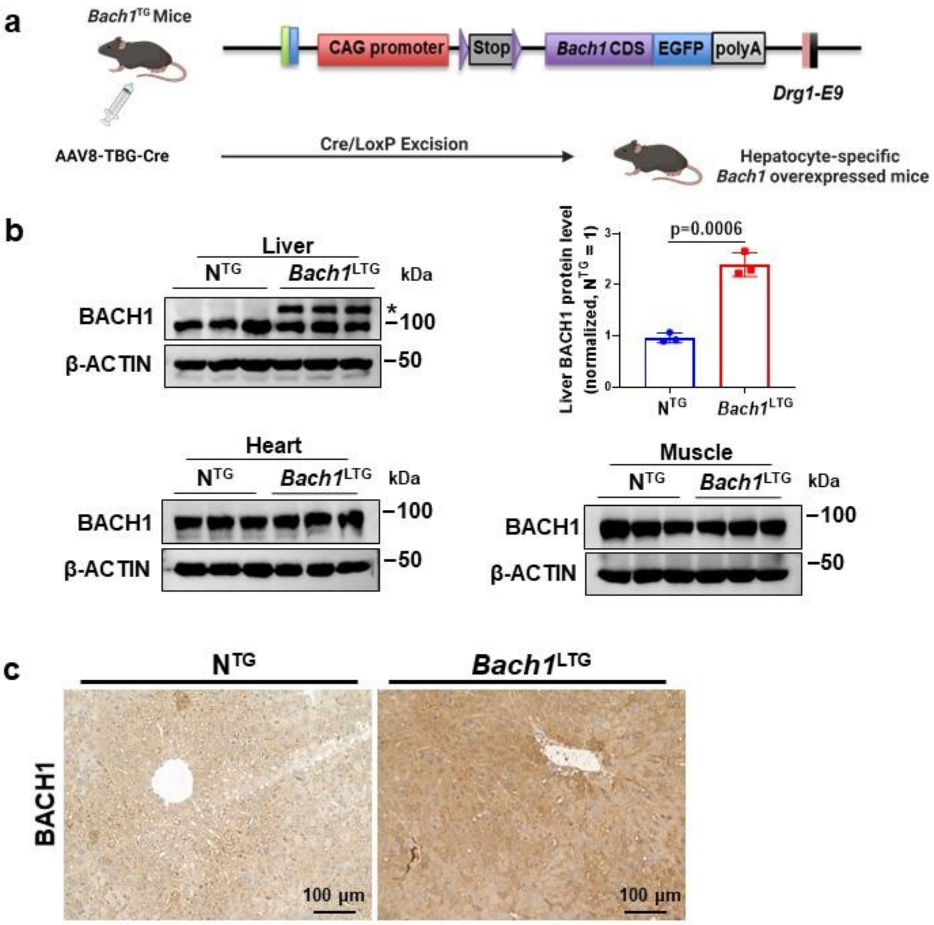

Fig. S4. BACH1 is specifically overexpressed in the mouse livers.

(a) A schematic diagram depicting the generation of hepatocyte-specific *Bach1* overexpressed mice. Fig. S4a was created with Biorender under paid subscription (Agreement number: JB262328TW). **(b)** Western blot analysis of BACH1 protein expression in the liver tissues (upper), heart (lower left), muscle (lower right) from *Bach1*^{LTG} and N^{TG} mice. Asterisks indicate exogenous BACH1 protein expression in the livers from *Bach1*^{LTG} mice. The columns show the quantification data of BACH1 protein levels of the mouse livers. Statistical analysis was performed by unpaired two-tailed Student's t-test (n=3 mice per group). **(c)** The representative images of immunohistochemistry assay of BACH1 in N^{TG} and *Bach1*^{LTG} mouse liver samples. Scale bar=100 μm. Data are presented as mean values ± SD. Source data are provided as a Source Data file.

Supplementary Fig 5

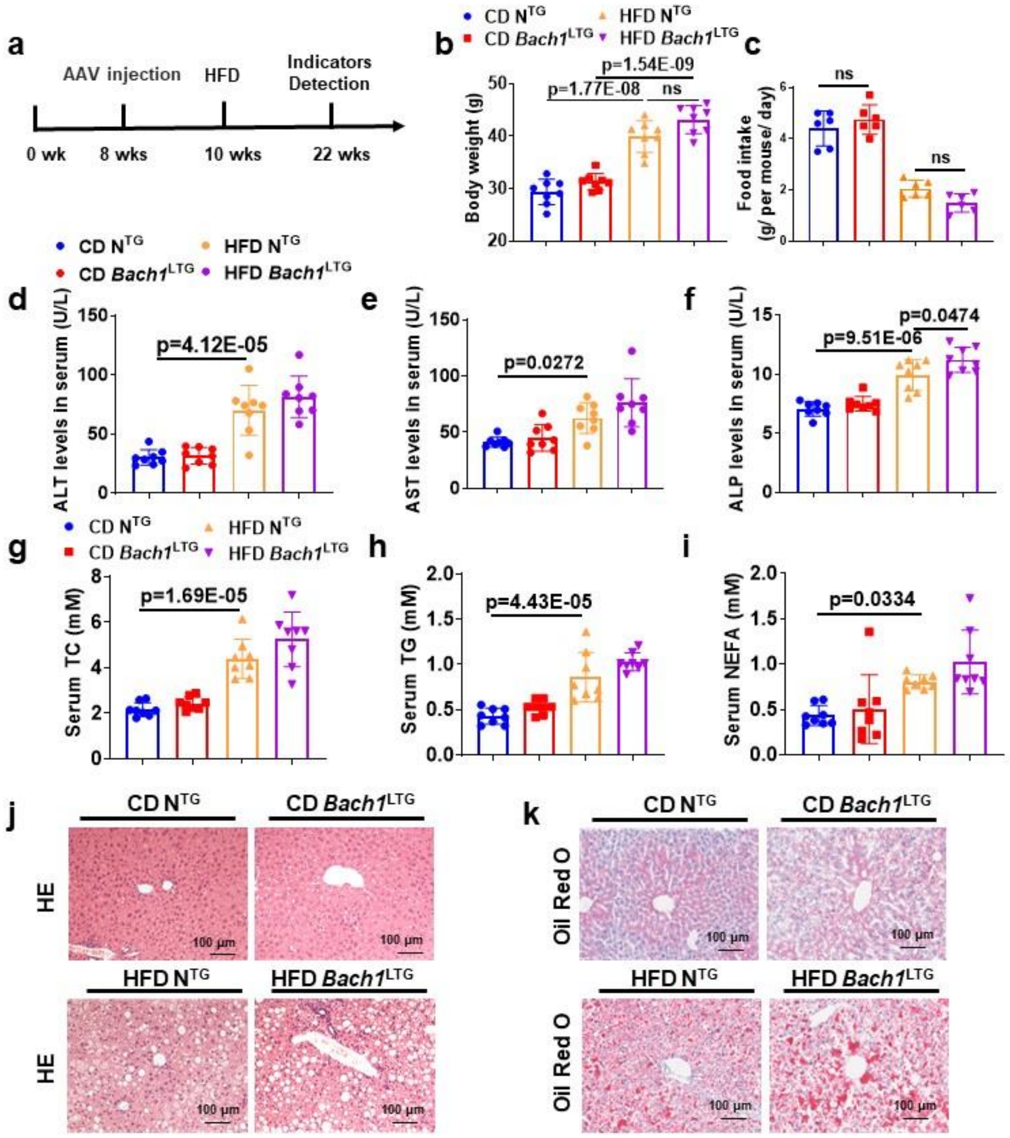

Fig.S5. Hepatic overexpression of BACH1 facilitates HFD-induced hepatic steatosis.

(a) Timeline of mouse modeling. **(b)** Body weight (n=8 mice per group) and **(c)** Food intake (n=6 mice per group). **(d-f)** Liver function manifested by serum ALT**(d)**, AST**(e)**, and ALP levels**(f)** of *Bach1*^{LTG} and N^{TG} mice fed a CD or HFD for 12 weeks. **(g-i)** TC**(g)**, TG**(h)**, and NEFA levels **(i)** were detected in the serum of N^{TG} and *Bach1*^{LTG} mice fed for 12 weeks with a CD or HFD (n=8 mice per group). **(j)** H&E and **(k)** Oil red O staining of liver tissues from N^{TG} and *Bach1*^{LTG} mice fed for 12 weeks with CD or HFD (n=8 mice per group). Scale bar=100 μm. Statistical analysis was performed by two-way ANOVA followed by Tukey post hoc tests for b, c, d, e, f, g, and h, by two-way ANOVA followed by Kruskal-Wallis test with Dunn multiple comparisons test for i. Data are presented as mean values ± SD. Source data are provided as a Source Data file.

Supplementary Fig 6

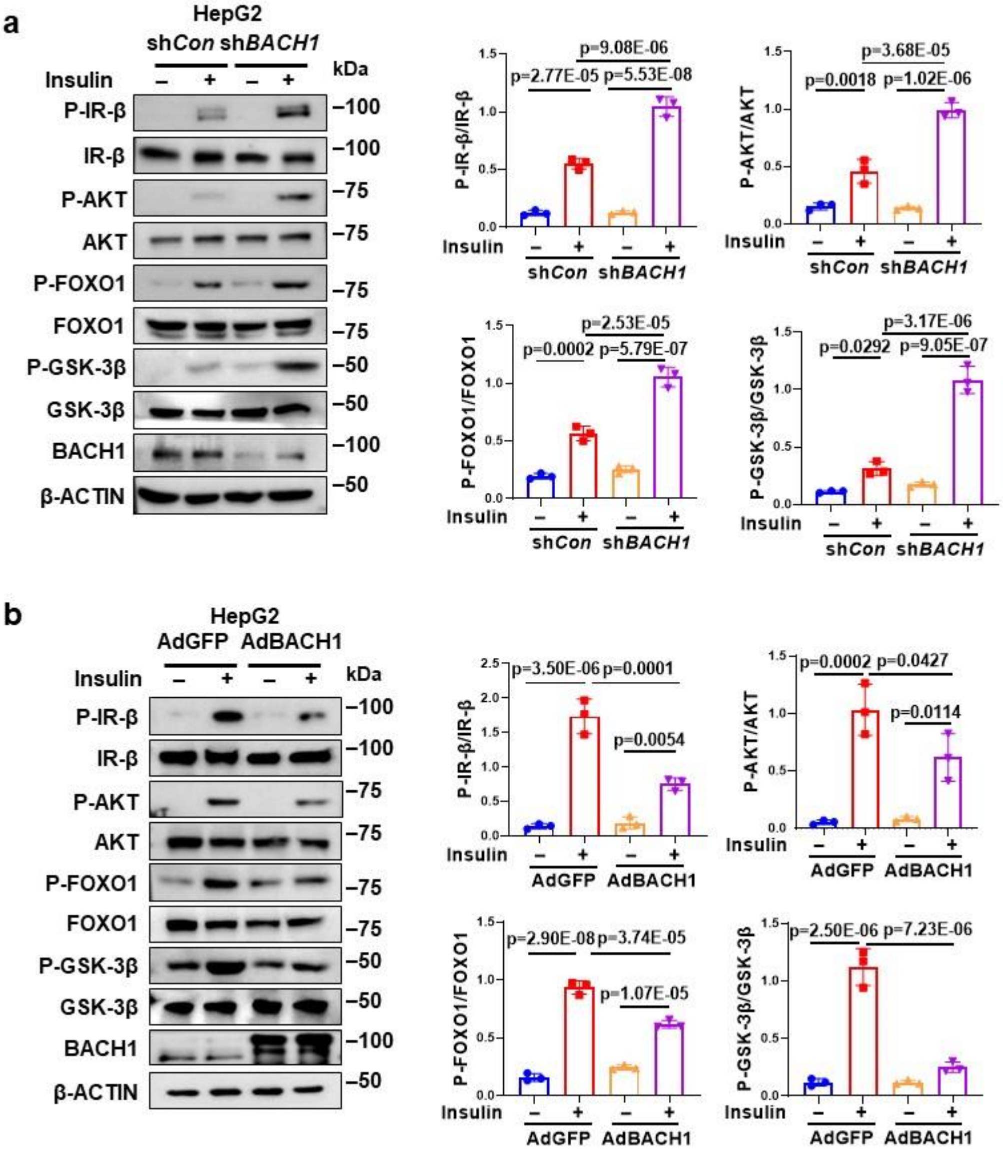

Fig.S6. BACH1 regulates insulin signaling in HepG2.

(a) Phosphorylated key molecules of the insulin pathway in HepG2 cells infected with sh*Con* or sh*BACH1* and then stimulated with insulin (100 nM) for 10 minutes were determined by Western blot. The right column panels are phosphorylated protein levels normalized to total protein (n = 3 biological replicates). **(b)** Phosphorylation of key molecules of the insulin pathway was determined in the HepG2 cells infected with adenoviruses encoding BACH1 (AdBACH1) or GFP (AdGFP) and then stimulated with insulin (100 nM) for 10 minutes. The right column panels are phosphorylated protein levels normalized to total protein (n = 3 biological replicates). Statistical analysis was performed by two-way ANOVA followed by Tukey post hoc tests for a and b. Data are presented as mean values ± SD. Source data are provided as a Source Data file.

Supplementary Fig 7

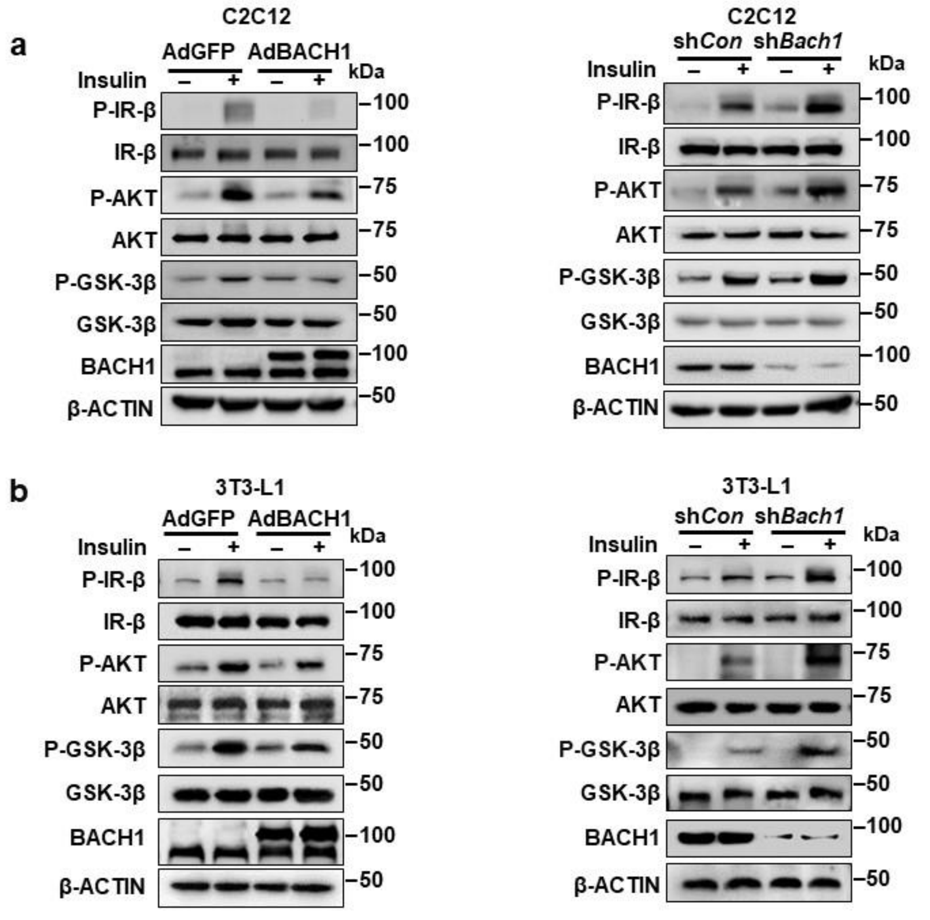

Fig.S7. BACH1 regulates insulin signaling in C2C12 and 3T3-L1 cells.

(a) Western blot analysis to detect phosphorylation of key molecules of the insulin pathway in C2C12 cells infected with adenovirus vector coding BACH1 (AdBACH1) (left) or shRNA targeting BACH1 (sh*Bach1*) (right) (n = 3 biological replicates). **(b)** Western blot analysis to detect phosphorylated key molecules of the insulin pathway in 3T3-L1 cells infected with AdBACH1 (left) or sh*Bach1*(right). Cells were infected with adenovirus for 48 h and then stimulated with insulin (100 nM) for 10 minutes (n = 3 biological replicates). Source data are provided as a Source Data file.

Supplementary Fig 8

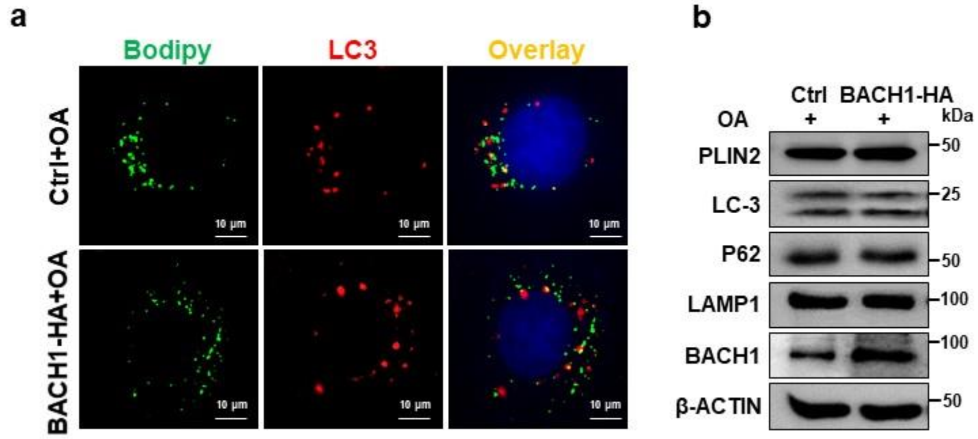

Fig. S8. Lipophagy was not affected by BACH1.

(a) Representative images of lipid droplets (LDs) by BODIPY staining (green) and autophagosomes by LC3 staining (red) in HepG2 cells with or without BACH1 overexpression after treated with 1 mM OA for 24 h. Nuclei were stained with DAPI (blue) (n = 3 biological replicates). Scale bar=10 μm. **(b)** Western blot analysis of lipophagy-associated protein. Source data are provided as a Source Data file (n = 3 biological replicates).

Supplementary Fig 9

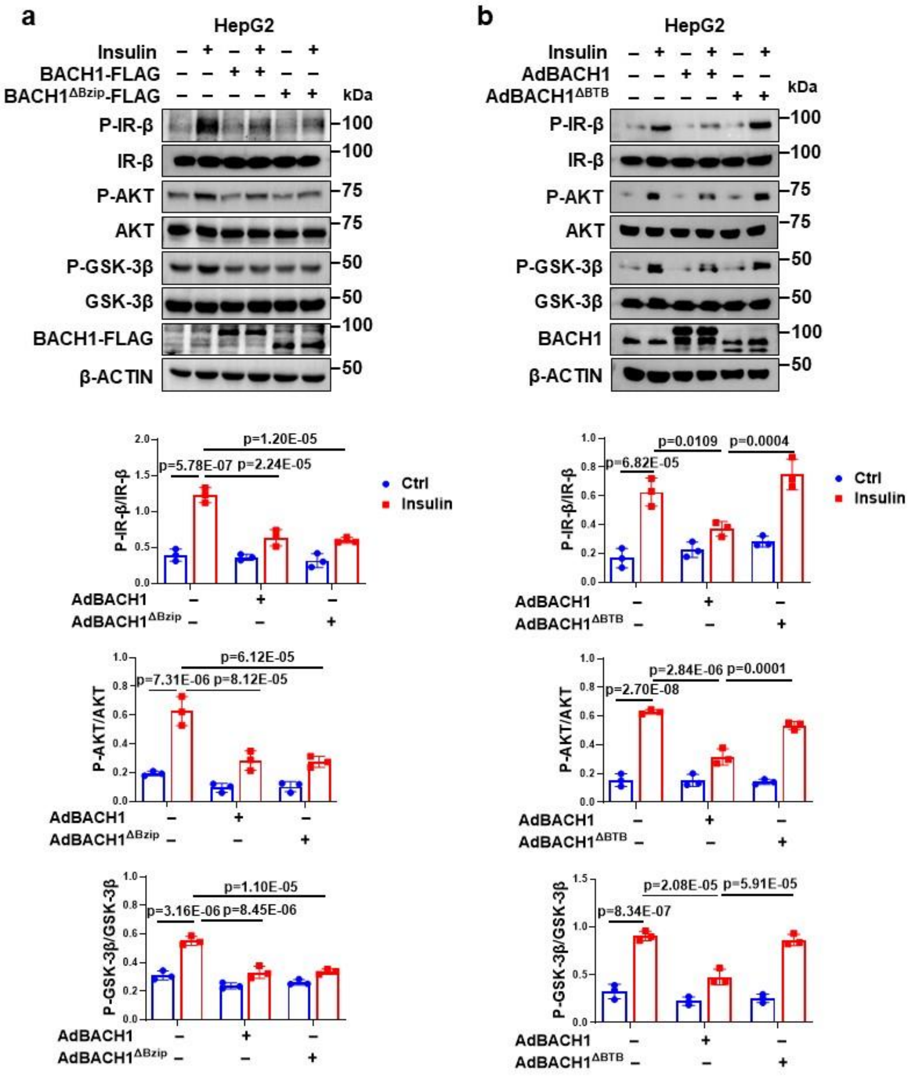

Fig. S9. BACH1 regulates insulin signaling with the BTB domain.

(a) Upper: Western blot analysis of the phosphorylation and total protein levels of IR-β, AKT, and GSK-3β in HepG2 cells transfected with vectors coding for flag-tagged versions of the full *Bach1* sequence or mutant sequences lacking the Bzip domain after treated with insulin (100 nM, 10 min) or not. Lower: Phosphorylated protein levels were normalized to total protein (n = 3 biological replicates). **(b)** Upper: Western blotting was performed to analyze the phosphorylation and total protein levels of IR-β, AKT, and GSK-3β in HepG2 cells infected with adenoviruses encoding AdBACH1 or AdBACH1$^{\Delta BTB}$ after being treated with or without insulin (100 nM, 10 min) in HepG2 cells. Lower: Phosphorylated protein levels were normalized to total protein (n = 3 biological replicates). Statistical analysis was performed by two-way ANOVA followed by Tukey post hoc tests for a and b. Data are presented as mean values ± SD. Source data are provided as a Source Data file.

Supplementary Fig 10

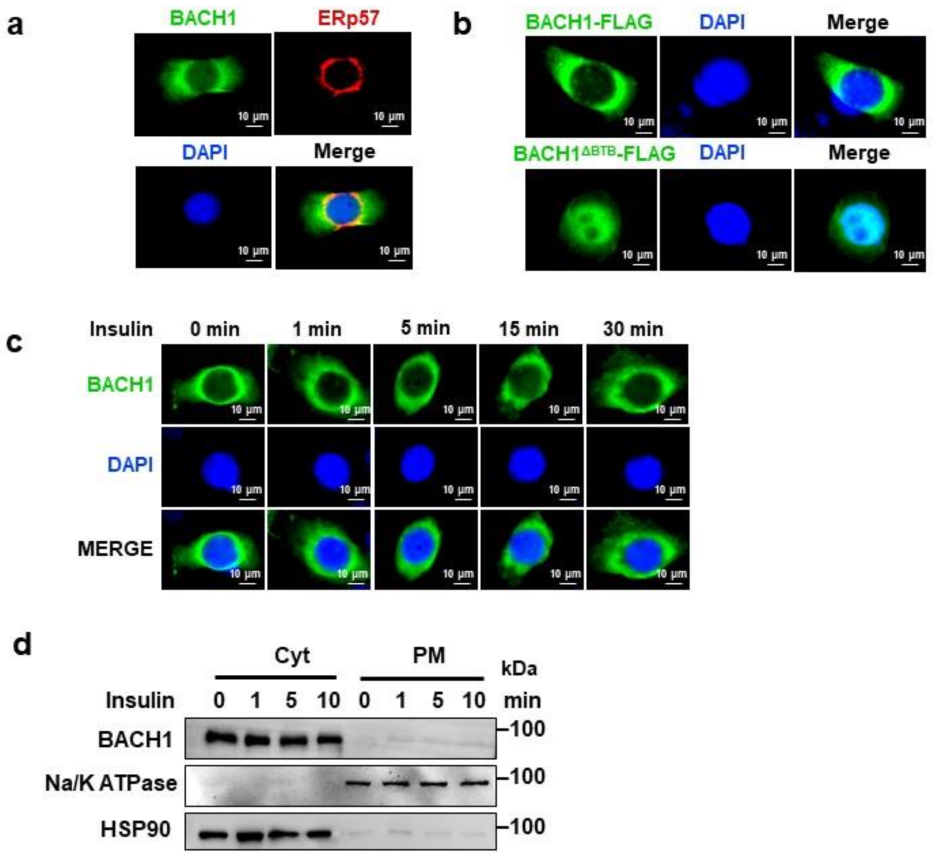

Fig. S10. BACH1 ER localization is not affected by insulin stimulation but by the BTB domain.

(a) Representative immunostaining of BACH1 (green) and ERp57 (red) in HepG2 cells. HepG2 cells were fixed and stained with anti-BACH1 and anti-ERp57 antibodies (n = 3 biological replicates). Scale bar=10 μm. **(b)** HepG2 cells were transfected with BACH1-FLAG or BACH1$^{\Delta BTB}$-FLAG for 48 h and then fixed and stained with an anti-FLAG antibody (green) (n = 3 biological replicates). Scale bar=10 μm. **(c)** Representative immunostaining of BACH1 (green) in HepG2 cells upon insulin stimulation. HepG2 cells were treated with 100 nM insulin for 0 min, 1 min, 5 min, 15 min, and 30 min respectively, and then fixed and stained with anti-BACH1 antibody. Nuclei of a-c were stained with DAPI (blue) (n = 3 biological replicates). Scale bar=10 μm. **(d)** Western blot analysis of BACH1 in the cytoplasm and plasma membrane of mouse primary hepatocytes. Cells were treated with 100 nM insulin for 0 min, 1 min, 5 min, and 10 min, and then the plasma membrane (PM) and cytosol (Cyt) were fractionated and analyzed by immunoblotting (n = 3 biological replicates). The Na/K ATPase and HSP90 were used as PM and Cyt loading controls, respectively. Source data are provided as a Source Data file.

Supplementary Fig 11

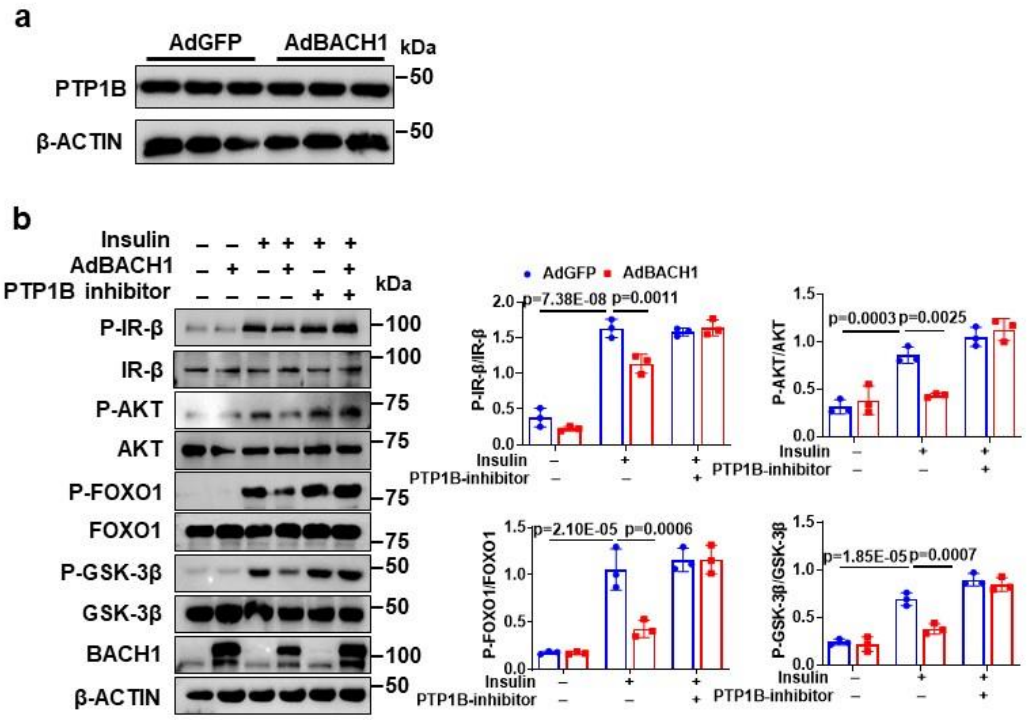

Fig.S11. PTP1B inhibitor attenuates BACH1-mediated suppression of insulin signaling. **(a)** PTP1B protein level was detected by western blot in HepG2 cells infected with AdBACH1 or AdGFP (n = 3 biological replicates). **(b)** Western blotting was performed to analyze the phosphorylation and total protein levels of key molecules of insulin signaling including IR-β, AKT, FOXO1, and GSK-3β in the primary hepatocytes infected with AdBACH1 or AdGFP and then treated with PTP1B inhibitor (10 μM, 4 h) in the presence or absence of insulin (100 nM, 10 min). The right column panels are phosphorylated protein levels normalized to total protein (n = 3 biological replicates). Statistical analysis was performed by two-way ANOVA followed by Tukey post hoc tests for b. Data are presented as mean values ± SD. Source data are provided as a Source Data file.

Supplementary Fig 12

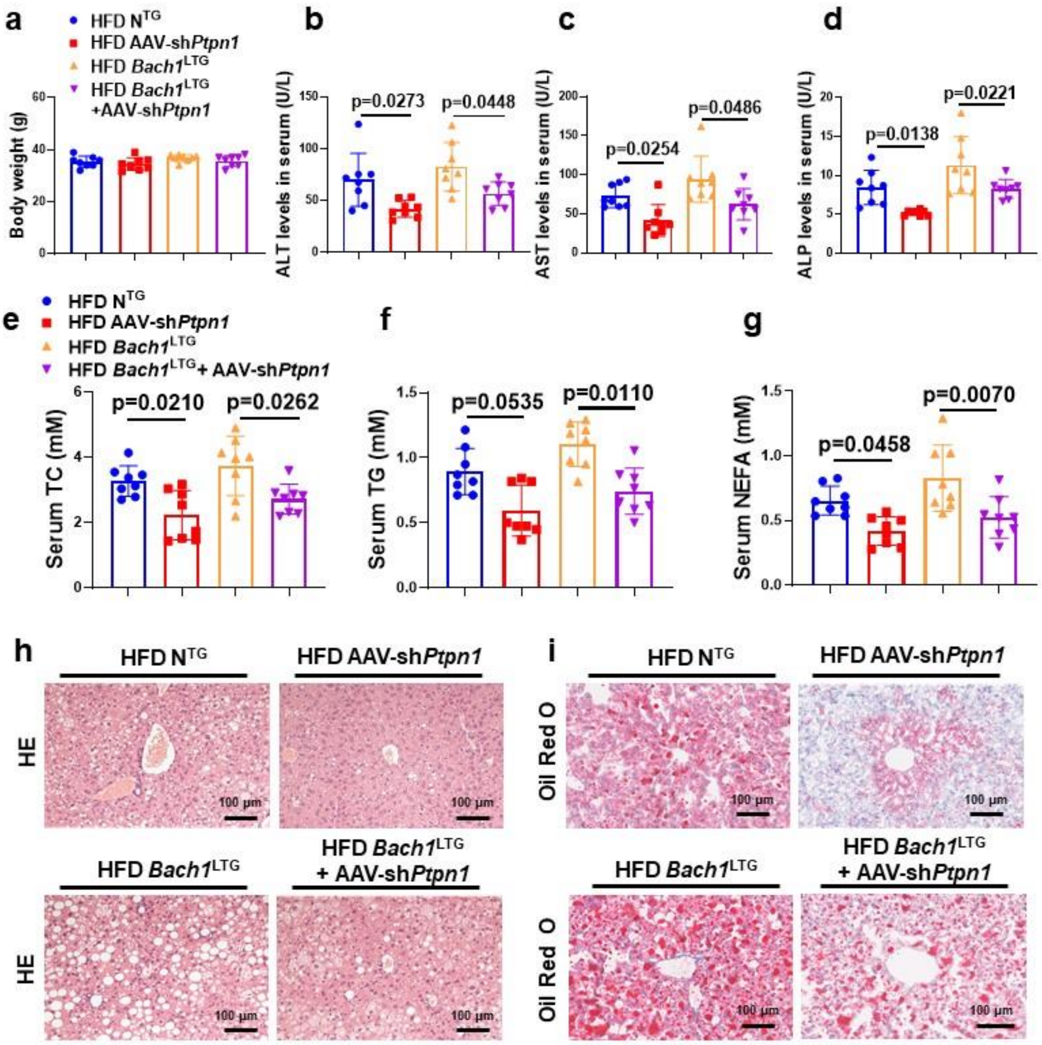

Fig.S12. BACH1 facilitates HFD-induced hepatic steatosis in a PTP1B-dependent manner. **(a)** Body weight (n=8 mice per group). **(b-d)** Liver function manifested by serum ALT**(b)**, AST**(c)**, and ALP levels **(d)**. **(e-g)** TC **(e)**, TG **(f)**, and NEFA levels **(g)** were detected in the serum of N^{TG}, AAV-sh*Ptpn1*, *Bach1*^{LTG}, *Bach1*^{LTG}+ AAV-sh*Ptpn1* mice fed for 12 weeks with HFD (n=8 mice per group). **(h)** H&E and **(i)** Oil red O staining of liver tissues from N^{TG}, AAV-sh*Ptpn1*, *Bach1*^{LTG}, *Bach1*^{LTG}+AAV-sh*Ptpn1* mice fed for 12 weeks with HFD (n=8 mice per group). Scale bar=100 µm. Statistical analysis was performed by two-way ANOVA followed by Tukey post hoc tests for a, b, e, g, by two-way ANOVA followed by Kruskal-Wallis test with Dunn multiple comparisons test for c, d, and f. Data are presented as mean values ± SD. Source data are provided as a Source Data file.

Supplementary Fig 13

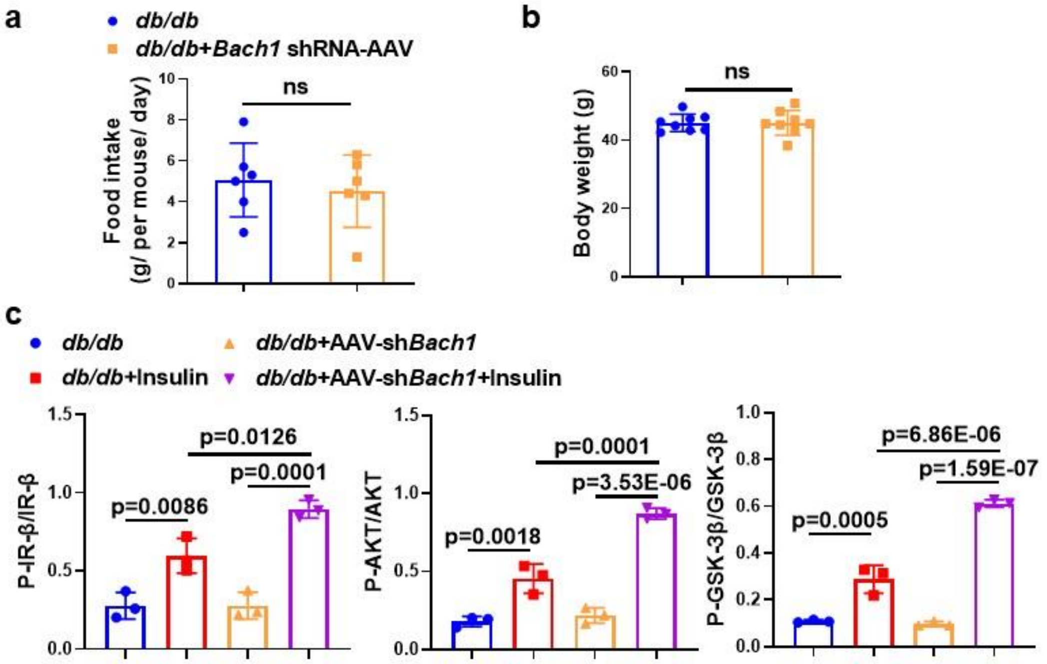

Fig.S13. Knockdown of BACH1 improves insulin signaling in *db/db* diabetic mice.

(a) Food intake (n=6 mice per group) and **(b)** Body weight (n=8 mice per group) of male *db/db* mice injected with AAV control or AAV-sh*Bach1* were examined. **(c)** The quantification data of Fig 8I were shown (n=3 mice per group). Phosphorylated protein levels were normalized to total protein. Statistical analysis was performed by unpaired two-tailed Student's t-test for a and b, by two-way ANOVA followed by Tukey post hoc tests for c. Data are presented as mean values ± SD. Source data are provided as a Source Data file.

Uncropped Scans of Blots in Supplementary Figures

Supplementary Fig.1g

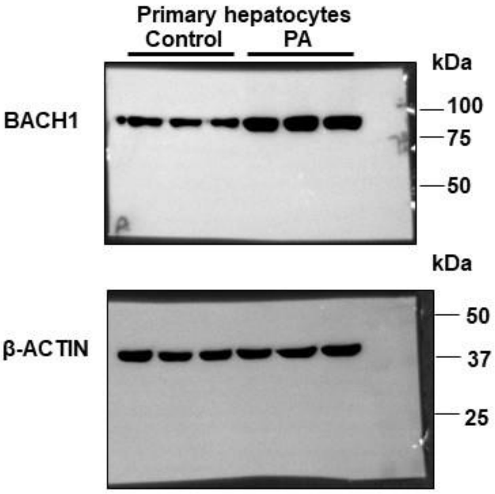

Supplementary Fig. 2b

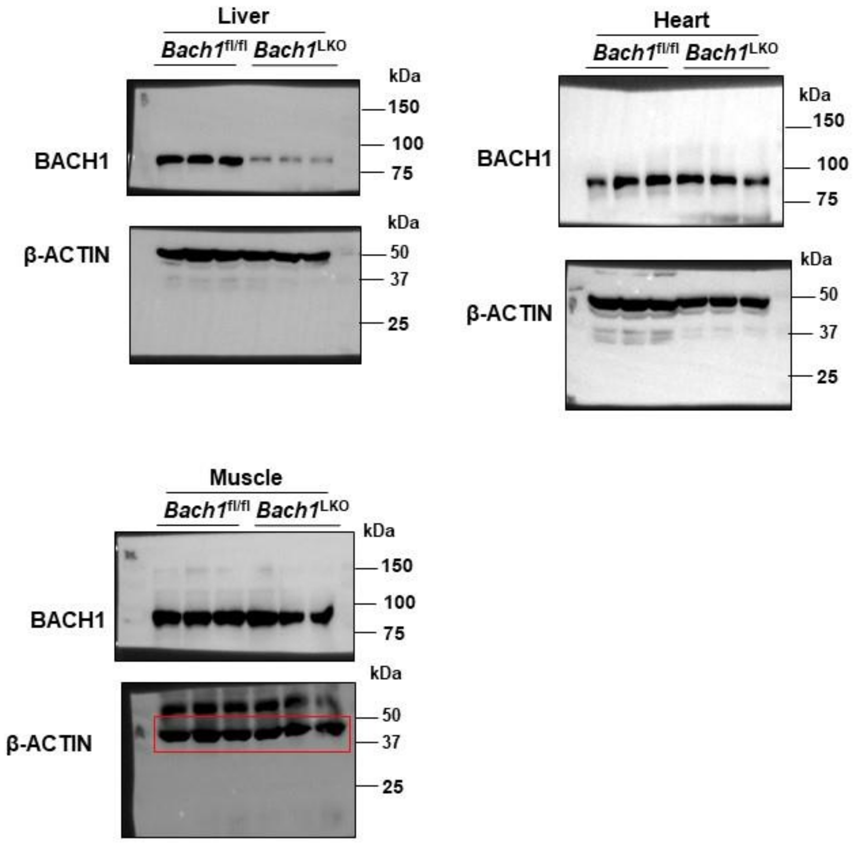

Supplementary Fig. 4b

Asterisks indicate exogenous BACH1 protein expression in the livers from *Bach1*LTG mice.

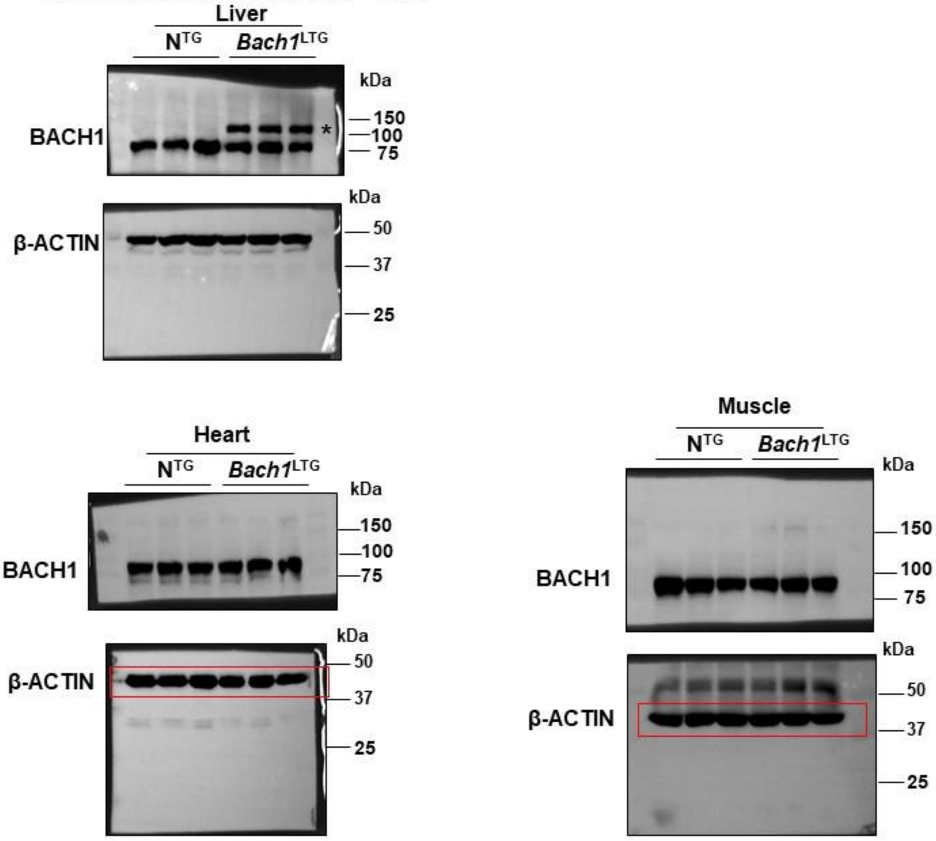

Supplementary Fig. 6a

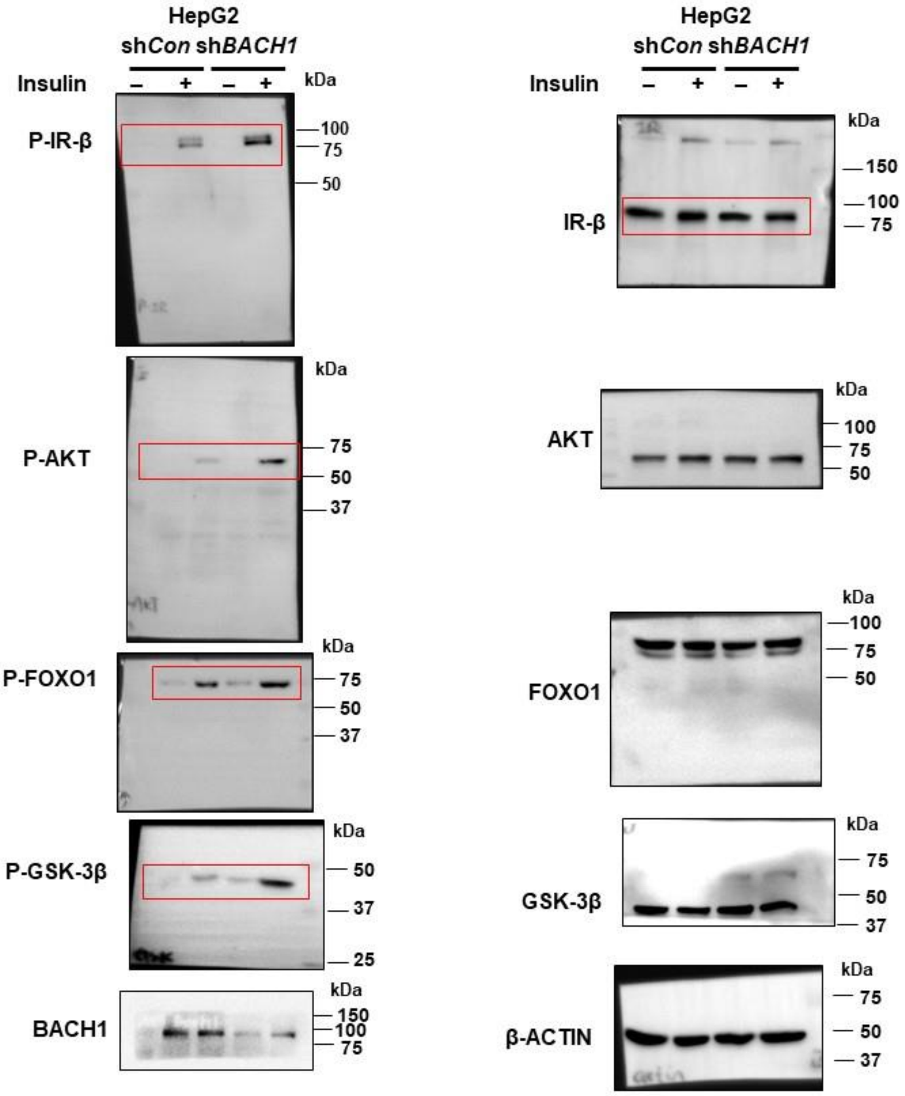

Supplementary Fig. 6b

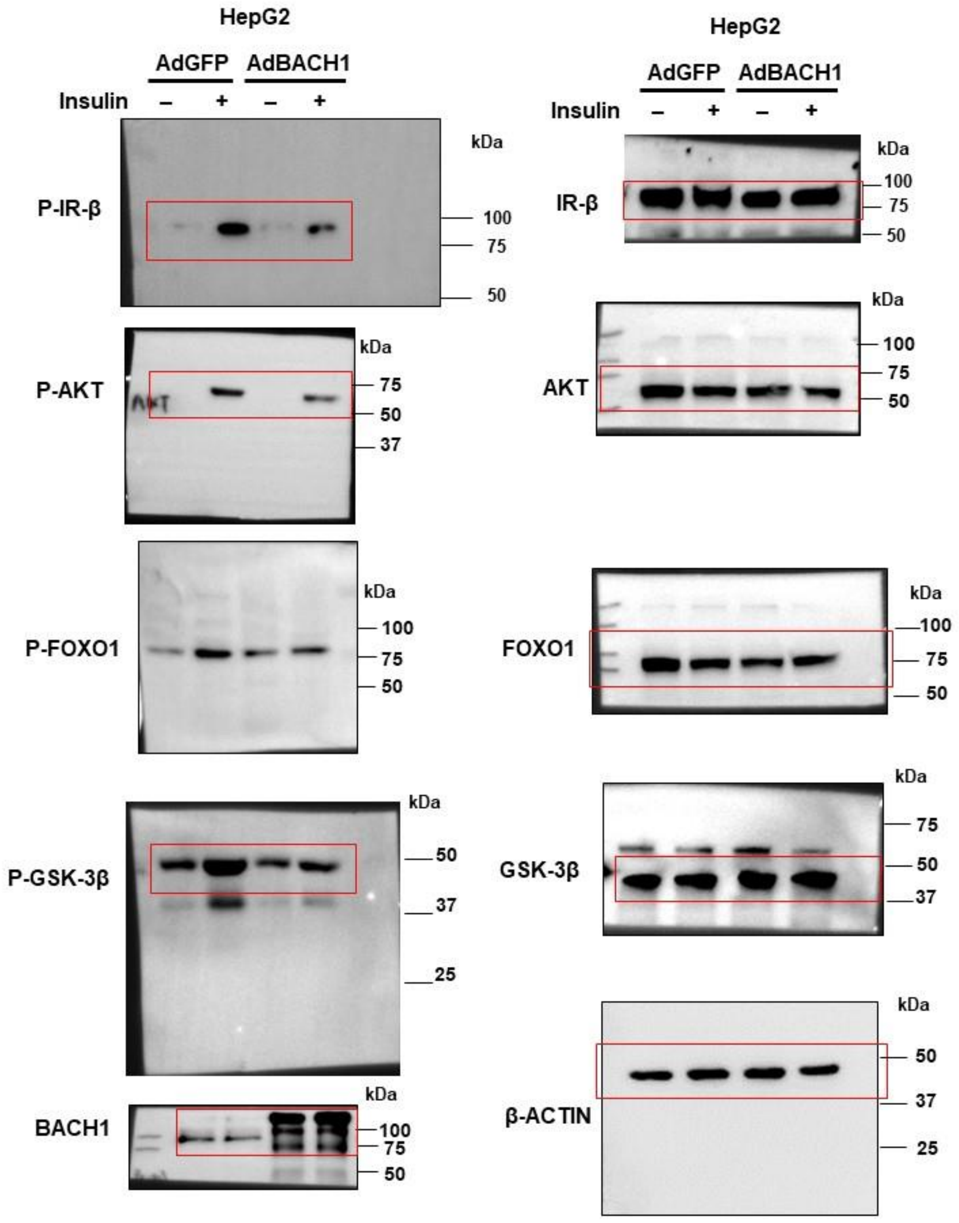

Supplementary Fig. 7a (Left)

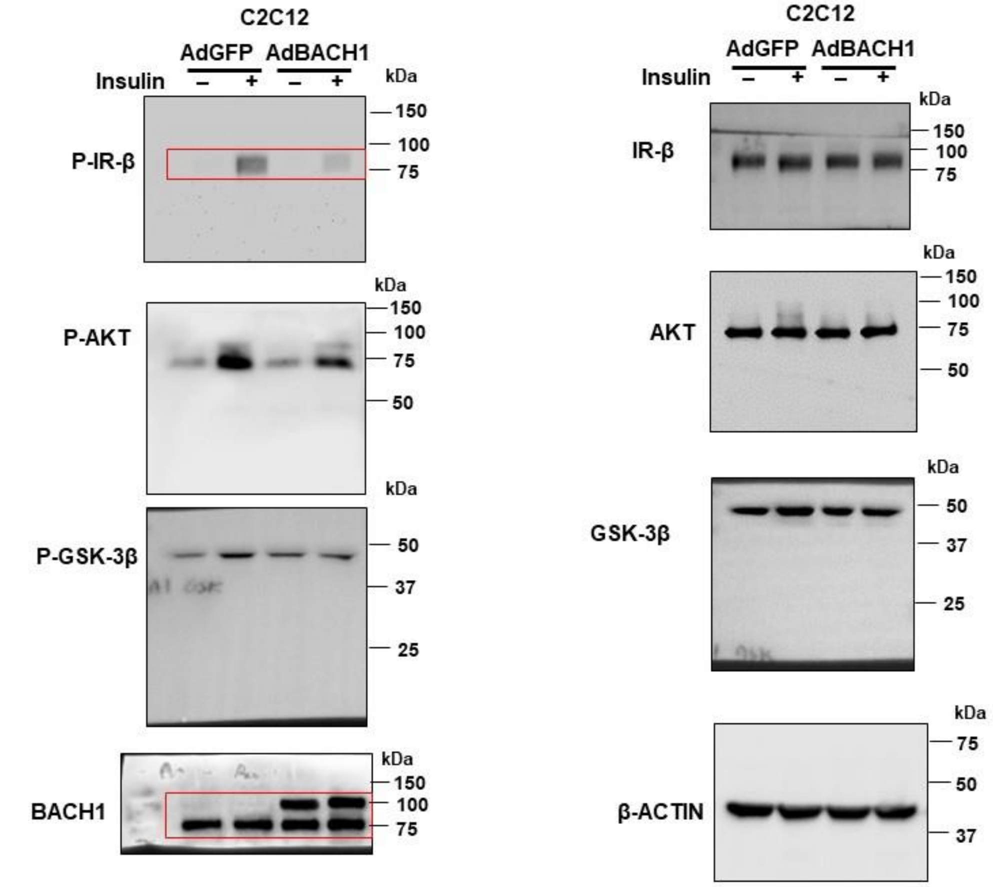

Supplementary Fig. 7a (Right)

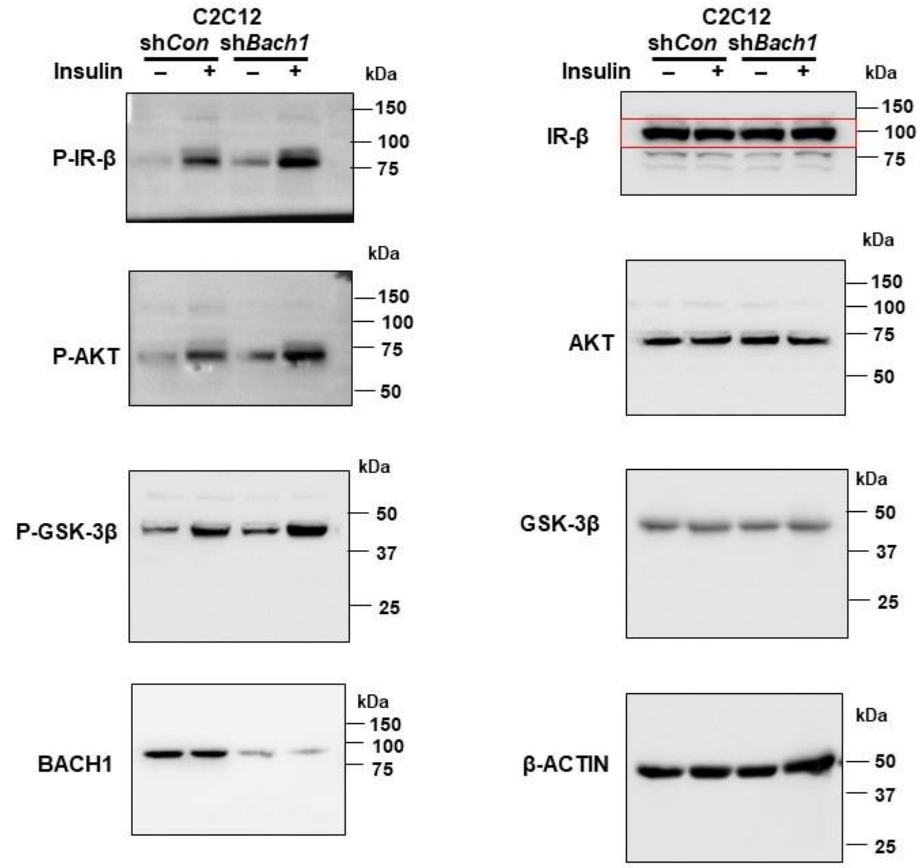

Supplementary Fig. 7b(Left)

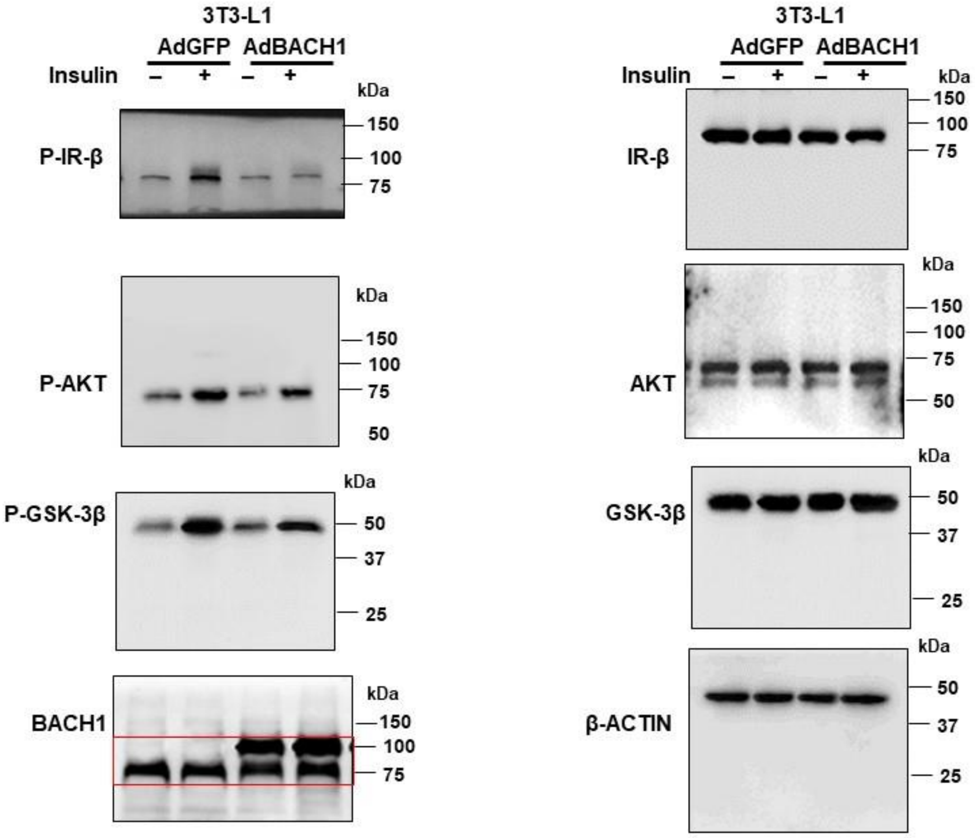

Supplementary Fig. 7b (Right)

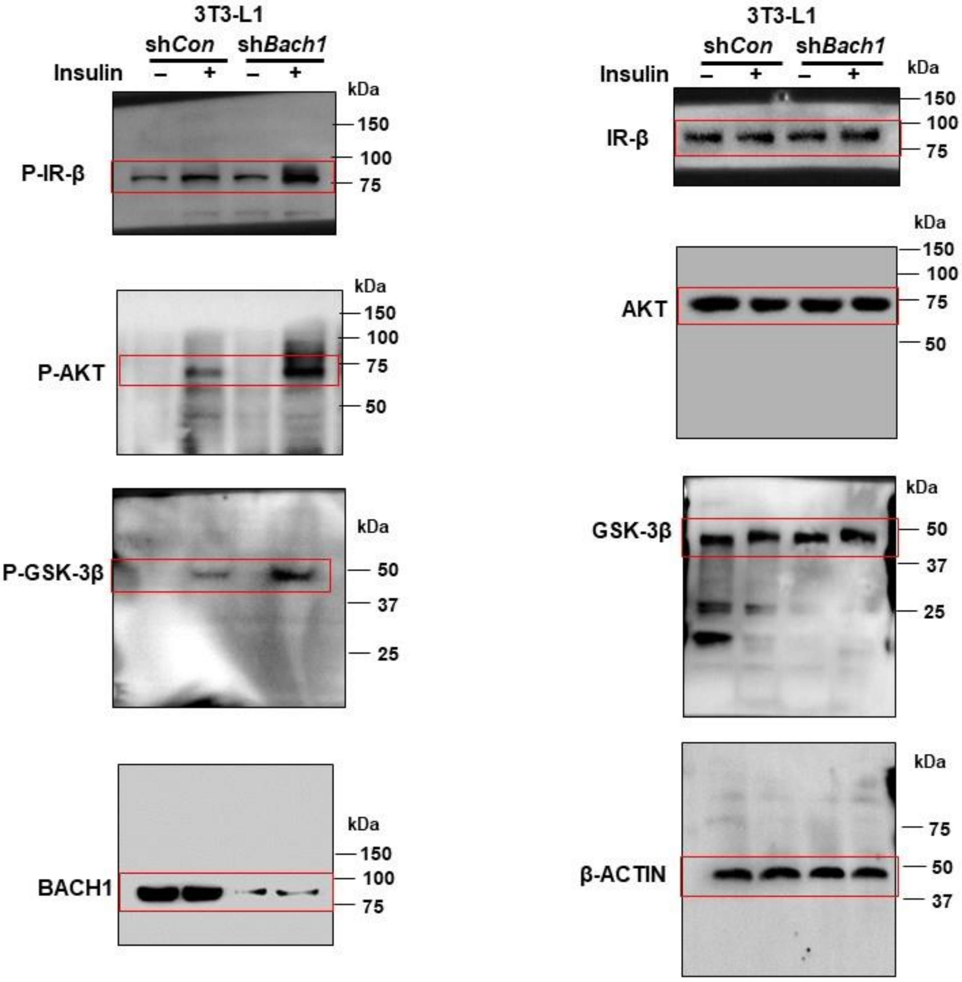

Supplementary Fig. 8b

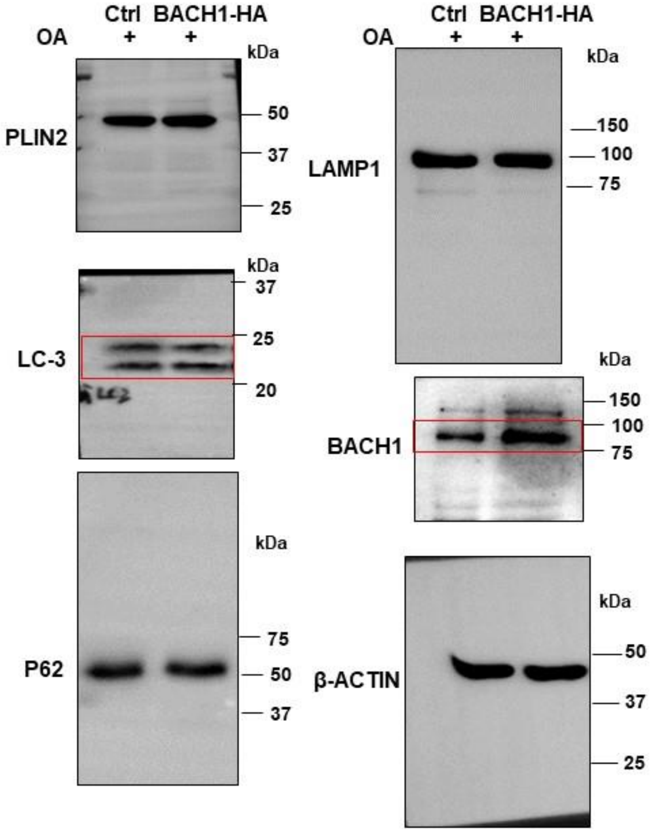

Supplementary Fig.9a

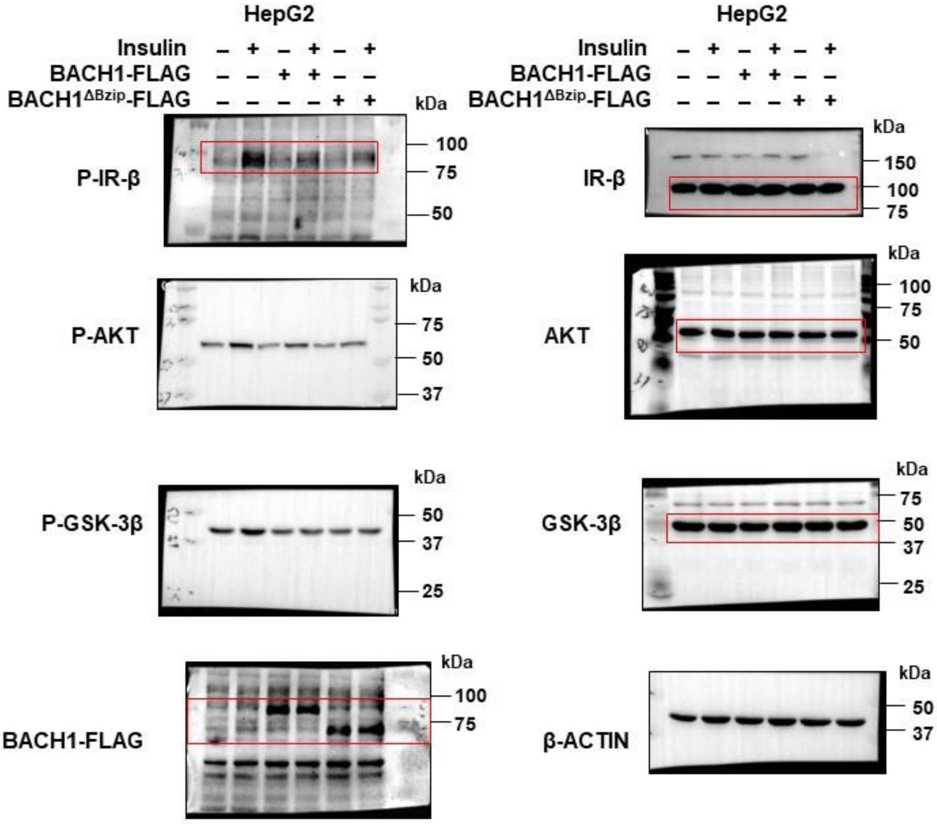

Supplementary Fig.9b

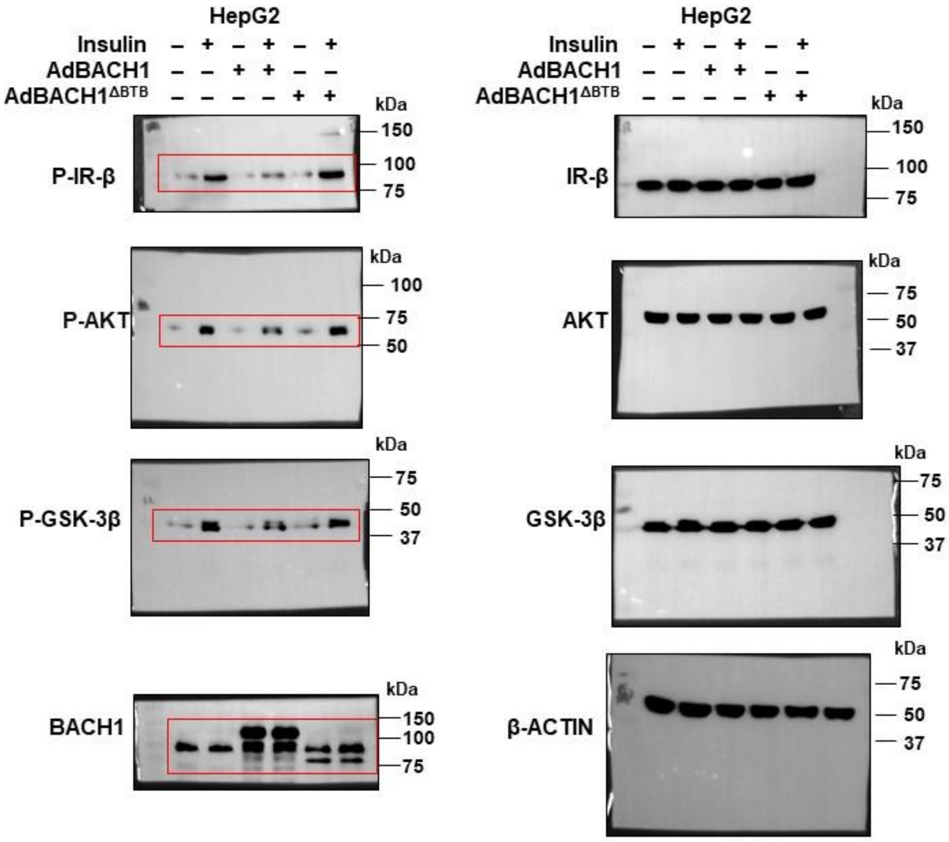

Supplementary Fig.10d

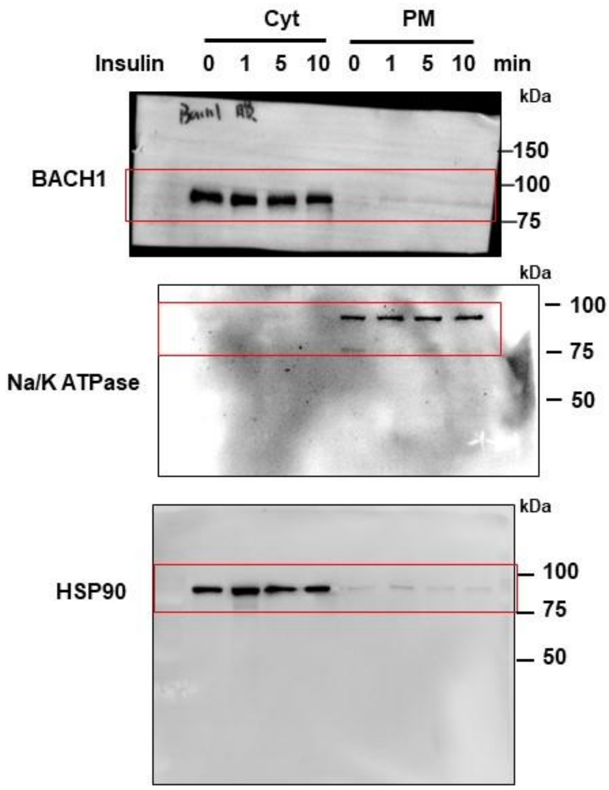

Supplementary Fig. 11a

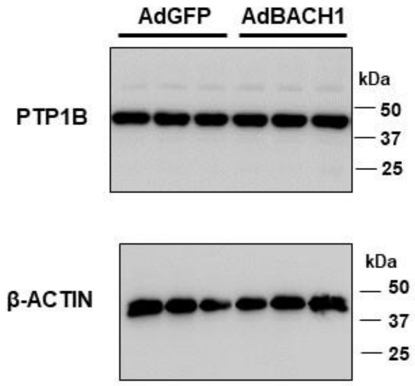

Supplementary Fig. 11b

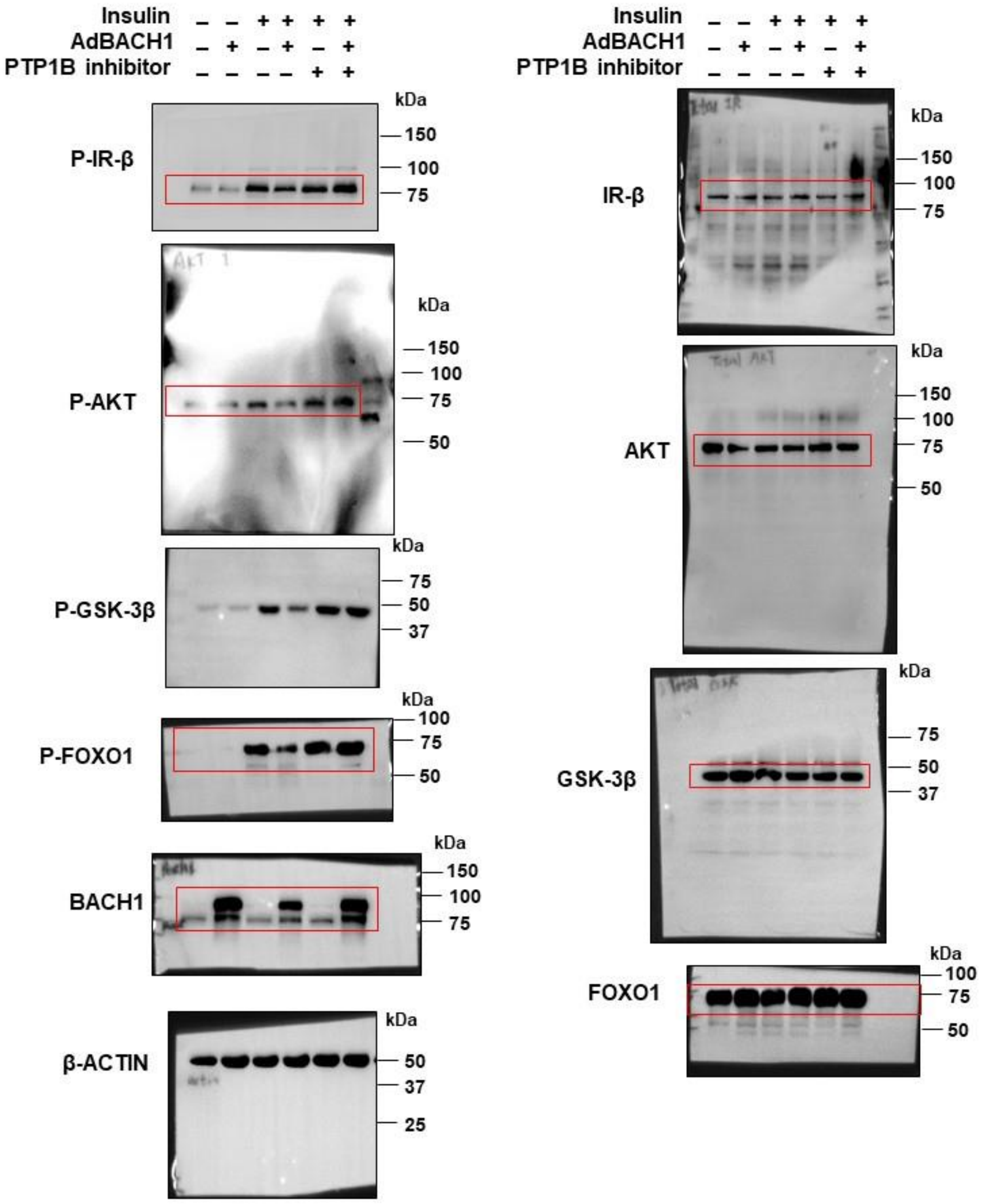

Supplement: Supplementary file 1 — Supplementary Information [file 41467_2023_44088_MOESM1_ESM.pdf]
