# Supplementary material for: BACH1 controls hepatic insulin signaling and glucose homeostasis in mice
Source: Nat Commun. 2023 Dec 21;14:8428. doi: 10.1038/s41467-023-44088-z (PMC10739811; doi:10.1038/s41467-023-44088-z)
Supplement: Supplementary file 3 — Reporting Summary [file 41467_2023_44088_MOESM3_ESM.pdf]

nature portfolio

Corresponding author(s): Dan Meng

Last updated by author(s): Oct 15, 2023

Reporting Summary

Nature Portfolio wishes to improve the reproducibility of the work that we publish. This form provides structure for consistency and transparency in reporting. For further information on Nature Portfolio policies, see our Editorial Policies and the Editorial Policy Checklist.

Statistics

For all statistical analyses, confirm that the following items are present in the figure legend, table legend, main text, or Methods section.

n/a	Confirmed	
☐	☒	The exact sample size (*n*) for each experimental group/condition, given as a discrete number and unit of measurement
☐	☒	A statement on whether measurements were taken from distinct samples or whether the same sample was measured repeatedly
☐	☒	The statistical test(s) used AND whether they are one- or two-sided<br>*Only common tests should be described solely by name; describe more complex techniques in the Methods section.*
☐	☒	A description of all covariates tested
☐	☒	A description of any assumptions or corrections, such as tests of normality and adjustment for multiple comparisons
☐	☒	A full description of the statistical parameters including central tendency (e.g. means) or other basic estimates (e.g. regression coefficient) AND variation (e.g. standard deviation) or associated estimates of uncertainty (e.g. confidence intervals)
☐	☒	For null hypothesis testing, the test statistic (e.g. *F*, *t*, *r*) with confidence intervals, effect sizes, degrees of freedom and *P* value noted<br>*Give P values as exact values whenever suitable.*
☒	☐	For Bayesian analysis, information on the choice of priors and Markov chain Monte Carlo settings
☒	☐	For hierarchical and complex designs, identification of the appropriate level for tests and full reporting of outcomes
☒	☐	Estimates of effect sizes (e.g. Cohen's *d*, Pearson's *r*), indicating how they were calculated

Our web collection on statistics for biologists contains articles on many of the points above.

Software and code

Policy information about availability of computer code

Data collection	Confocal Laser Scanning Microscope (Leica Microsystems GmbH, DMI6000B):collecting the immunofluorescence data.<br>Bio-Rad iQ5 real-time PCR thermal cycler: collecting qRT-PCR data.<br>A gel imaging system (Tanon-4200, Shanghai, China): collecting Western-blot data.
Data analysis	The statistical analyses were performed by GraphPad Prism 8.0 Software (San Diego, CA, USA) and Image J 1.50b software (National Institutes of Health, Montgomery County, Maryland, United States).<br>Primer design was performed by Primer Premier 5.0 software.<br>The analyses of RNAseq data were performed by R Software (4.0.2).<br>The Figure 8j was created with Biorender.com under paid subscription.

For manuscripts utilizing custom algorithms or software that are central to the research but not yet described in published literature, software must be made available to editors and reviewers. We strongly encourage code deposition in a community repository (e.g. GitHub). See the Nature Portfolio guidelines for submitting code & software for further information.

Data

Policy information about availability of data

All manuscripts must include a data availability statement. This statement should provide the following information, where applicable:

- Accession codes, unique identifiers, or web links for publicly available datasets
- A description of any restrictions on data availability
- For clinical datasets or third party data, please ensure that the statement adheres to our policy

There are no restrictions on data availability. All data supporting the findings of this study are available within main text, supplementary information and Source data. Source data are provided with this paper. The RNAseq data of BACH1 mRNA expression in the liver tissue of lean or obese individuals is under the accession number GSE192742 and are available at the following URL: https://www.ncbi.nlm.nih.gov/geo/query/acc.cgi?acc=GSE192742. The RNAseq data of BACH1 mRNA expression in the liver tissue of mice following a high-sucrose-and-high-fat diet (HSD) is under the accession number GSE182365 and are available at the following URL: https://www.ncbi.nlm.nih.gov/geo/query/acc.cgi?acc=GSE182365. If needed, contact J.J. for original data described in the paper. Contact D.M. for requesting Bach1fl/fl and Bach1TG mouse strain, and all other plasmids or reagents described in this article. Source data are provided with this paper.

Research involving human participants, their data, or biological material

Policy information about studies with human participants or human data. See also policy information about sex, gender (identity/presentation), and sexual orientation and race, ethnicity and racism.

Reporting on sex and gender

An equal number of male and female human participants were used in our study.

Reporting on race, ethnicity, or other socially relevant groupings

For the detection of human liver samples (Figure 1B-D), male and female patients were recruited by Zhongshan Hospital (Shanghai, China), there is no potential bias for recruiting patient cohort.

Population characteristics

The demographic and clinical characteristics of the NAFLD patients involved in the Figure 1B-D were provided in Supplementary Table S1-3 as followed. We have amended the information by expressing the age of the patients as a range instead of the actual number so that individuals can no longer be identified.

Table S1-3. Information of the human liver samples

Symptom	Gender	Steatosis	Age
Normal-1	Male	No	
Normal-2	Male	No	median age 41
Normal-3	Male	No	(range 28-67)
Normal-4	Female	No	
Normal-5	Female	No	
Normal-6	Female	No	
NAFLD-1	Male	40% steatosis	
NAFLD-2	Male	40% steatosis	
NAFLD-3	Male	10% steatosis	median age 36
NAFLD-4	Female	50% steatosis	(range 26-54)
NAFLD-5	Female	20% steatosis	
NAFLD-6	Female	15% steatosis	

Recruitment

Patients in this study were recruited from Zhongshan Hospital, Shanghai, China. All patients information and samples were collected based on clinical requirement for diagnosis. There were no self-selection bias or other biases.

Ethics oversight

The study protocol was approved by the Ethics Committee Board at the School of Basic Medical Sciences, Fudan University (approval number: 2016-002). Each patient provided written informed consent. All procedures complied with the ethical guidelines of the Declaration of Helsinki.

Note that full information on the approval of the study protocol must also be provided in the manuscript.

Field-specific reporting

Please select the one below that is the best fit for your research. If you are not sure, read the appropriate sections before making your selection.

☒ Life sciences ☐ Behavioural & social sciences ☐ Ecological, evolutionary & environmental sciences

For a reference copy of the document with all sections, see nature.com/documents/nr-reporting-summary-flat.pdf

Life sciences study design

All studies must disclose on these points even when the disclosure is negative.

Sample size

Sample size was determined based on statistical requirements and previous published studies using similar methodologies in this field (ref1,2,3). For in vivo studies, the sample size was determined to be enough to obtain the statistical difference between groups, and genotype. Animals were randomly assigned to treatment groups. All sample sizes are listed in the corresponding figure legends or on the figures. All experiments were repeated at least three times.

REFERENCE:
1.Qiao A., et al. Sam68 promotes hepatic gluconeogenesis via CRTC2. Nat Commun. 2021 Jun 7;12(1):3340. doi: 10.1038/s41467-021-23624-9. PMID: 34099657; PMCID: PMC8185084.
2.Guo X., et al. Regulation of age-associated insulin resistance by MT1-MMP-mediated cleavage of insulin receptor. Nat Commun. 2022 Jun 29;13(1):3749. doi: 10.1038/s41467-022-31563-2. PMID: 35768470; PMCID: PMC9242991.
3.Yao Z., et al. Upregulation of WDR6 drives hepatic de novo lipogenesis in insulin resistance in mice. Nat Metab. 2023 Sep 21. doi: 10.1038/s42255-023-00896-7. Epub ahead of print. PMID: 37735236.

Data exclusions	No data were excluded from the analyses.
Replication	All experiments were conducted at least three times independently, and similar results were adopted for further analysis to guarantee reproducibility.
Randomization	Samples were randomly allocated into the study.
Blinding	This study included a lot of complicated experimental design, the feasibility of blinding was poor, thus blinding was not efficiently applied. The investigators were blinded to group allocation during data collection. Data analysis were performed by different investigators and analysis to avoid conscious and unconscious bias.

Reporting for specific materials, systems and methods

We require information from authors about some types of materials, experimental systems and methods used in many studies. Here, indicate whether each material, system or method listed is relevant to your study. If you are not sure if a list item applies to your research, read the appropriate section before selecting a response.

Materials & experimental systems

n/a	Involved in the study	
☐	☒	Antibodies
☐	☒	Eukaryotic cell lines
☒	☐	Palaeontology and archaeology
☐	☒	Animals and other organisms
☒	☐	Clinical data
☒	☐	Dual use research of concern
☒	☐	Plants

Methods

n/a	Involved in the study	
☒	☐	ChIP-seq
☒	☐	Flow cytometry
☒	☐	MRI-based neuroimaging

Antibodies

Antibodies used

BACH1, Santa Cruz, sc-271211, diluted 1:200
BACH1, proteintech, 14018-1-AP, diluted 1:1000
β-ACTIN, Proteintech, 66009-1-lg, diluted 1:10000
IR-β, Cell Signaling Technology, 3025, diluted 1:1000
FLAG, Sigma-Aldrich, F1804, diluted 1:1000
HA, Santa Cruz, sc-7392, diluted 1:200
p-IR-β, Cell Signaling Technology, 3021, diluted 1:1000
p-AKT, Cell Signaling Technology, 4060, diluted 1:1000
AKT, Cell Signaling Technology, 4691, diluted 1:1000
p-GSK-3β, Cell Signaling Technology , 9322, diluted 1:1000
GSK-3β, Cell Signaling Technology, 12456, diluted 1:1000
p-FOXO1, Cell Signaling Technology, 2599, diluted 1:1000
FOXO1, Cell Signaling Technology, 2880, diluted 1:1000
PTP1B, Proteintech, 11334-1-AP, diluted 1:1000
PP2A-Aα, Santa Cruz, sc-56954, diluted 1:200
PTEN, Santa Cruz, sc-7974, diluted 1:200
GFP, Proteintech, 50430-2-AP, diluted 1:1000
LC3B, Cell Signaling Technology, 3868, diluted 1:1000
SQSTM1/p62, Cell Signaling Technology, 23214, diluted 1:1000
LAMP1, Cell Signaling Technology, 9091, diluted 1:1000
PLIN2 Abcam ab108323 WB 1:1000
Alexa-Fluor 488 Donkey Anti-Rabbit lgG, Jackson, 711-545-152, diluted 1:200
Alexa-Fluor 594 Donkey Anti-Rabbit lgG, Jackson, 711-585-152, diluted 1:200
Alexa-Fluor 488 Donkey Anti-Mouse lgG, Jackson, 715-545-150, diluted 1:200
Alexa-Fluor 647 Donkey Anti-Rabbit lgG, Abcam, ab150075, diluted 1:200
HRP-Goat Anti-Mouse lgG, Thermo Fisher Scientific, 31460, diluted 1:5000
HRP-Goat Anti-Rabbit lgG, Thermo Fisher Scientific, 31430, diluted 1:5000

ERp57/ERp60 Polyclonal antibody, Proteintech, 15967-1-AP, diluted 1:1000

Validation

All antibodies sourced from commercial corporation are well-validated by the manufacturer and are widely used in the scientific community, information and validation of antibodies used in this work are available on the manufacturers' websites listed below:

BACH1, Santa Cruz, sc-271211
https://www.scbt.com/p/bach1-antibody-f-9?requestFrom=search

BACH1, proteintech, 14018-1-AP
https://www.ptgcn.com/products/BACH1-Antibody-14018-1-AP.htm

β-ACTIN, Proteintech, 66009-1-lg
https://www.ptgcn.com/products/Pan-Actin-Antibody-66009-1-Ig.htm

IR-β, Cell Signaling Technology, 3025
https://www.cellsignal.com/products/primary-antibodies/insulin-receptor-b-4b8-rabbit-mab/3025

FLAG, Sigma-Aldrich, F1804
HA, Santa Cruz, sc-7392
https://www.scbt.com/p/ha-probe-antibody-f-7?requestFrom=search

p-IR-β, Cell Signaling Technology, 3021
https://www.cellsignal.com/products/primary-antibodies/phospho-igf-i-receptor-b-tyr1131-insulin-receptor-b-tyr1146-antibody/3021

p-AKT, Cell Signaling Technology, 4060
https://www.cellsignal.com/products/primary-antibodies/phospho-akt-ser473-d9e-xp-rabbit-mab/4060

AKT, Cell Signaling Technology, 4691
https://www.cellsignal.com/products/primary-antibodies/akt-pan-c67e7-rabbit-mab/4691

p-GSK-3β, Cell Signaling Technology , 9322
https://www.cellsignal.com/products/primary-antibodies/phospho-gsk-3-beta-ser9-d3a4-rabbit-mab/9322

GSK-3β, Cell Signaling Technology, 12456
https://www.cellsignal.com/products/primary-antibodies/gsk-3b-d5c5z-xp-rabbit-mab/12456

p-FOXO1, Cell Signaling Technology, 2599
https://www.cellsignal.com/products/primary-antibodies/phospho-foxo1-thr24-foxo3a-thr32-foxo4-thr28-4g6-rabbit-mab/2599

FOXO1, Cell Signaling Technology, 2880
https://www.cellsignal.com/products/primary-antibodies/foxo1-c29h4-rabbit-mab/2880

PTP1B, Proteintech, 11334-1-AP
https://www.ptgcn.com/products/PTPN1-Antibody-11334-1-AP.htm

PP2A-Aα, Santa Cruz, sc-56954
https://www.scbt.com/p/pp2a-aalpha-antibody-6g3?requestFrom=search

PTEN, Santa Cruz, sc-7974
https://www.scbt.com/p/pten-antibody-a2b1?requestFrom=search

GFP, Proteintech, 50430-2-AP
https://www.ptgcn.com/products/eGFP-Antibody-50430-2-AP.htm

LC3B, Cell Signaling Technology, 3868
https://www.cellsignal.com/products/primary-antibodies/lc3b-d11-xp-rabbit-mab/3868

SQSTM1/p62, Cell Signaling Technology, 23214
https://www.cellsignal.com/products/primary-antibodies/sqstm1-p62-d6m5x-rabbit-mab/23214

LAMP1, Cell Signaling Technology, 9091
https://www.cellsignal.com/products/primary-antibodies/lamp1-d2d11-xp-rabbit-mab/9091

PLIN2, Abcam, ab108323
https://www.abcam.cn/products/primary-antibodies/rabbit-monoclonal-epr3713-to-perilipin-2-ab108323.html

Alexa-Fluor 488 Donkey Anti-Rabbit IgG, Jackson, 711-545-152
https://www.jacksonimmuno.com/catalog/products/711-545-152

Alexa-Fluor 594 Donkey Anti-Rabbit lgG, Jackson, 711-585-152
https://www.jacksonimmuno.com/catalog/products/711-585-152

Alexa-Fluor 488 Donkey Anti-Mouse lgG, Jackson, 715-545-150
https://www.jacksonimmuno.com/catalog/products/715-545-150

Alexa-Fluor 647 Donkey Anti-Rabbit lgG, Abcam, ab150075
HRP-Goat Anti-Mouse lgG, Thermo Fisher Scientific, 31460
https://www.thermofisher.cn/cn/zh/antibody/product/Goat-anti-Rabbit-IgG-H-L-Secondary-Antibody-Polyclonal/31460

HRP-Goat Anti-Rabbit lgG, Thermo Fisher Scientific, 31430
https://www.thermofisher.cn/cn/zh/antibody/product/Goat-anti-Mouse-IgG-H-L-Secondary-Antibody-Polyclonal/31430

ERp57/ERp60 Polyclonal antibody, Proteintech, 15967-1-AP
https://www.ptgcn.com/products/PDIA3-Antibody-15967-1-AP.htm

Eukaryotic cell lines

Policy information about cell lines and Sex and Gender in Research

Cell line source(s)	Primary hepatocytes were isolated from 6- to 8-week-old male mice, HepG2 hepatocyte (Cat# TCHu 72), myoblast C2C12 (Cat# SCSP-505) and pre-adipocyte 3T3-L1 (Cat# SCSP-5038) were purchased from Chinese Academy of Sciences and cultured in DMEM supplemented with 10% FBS.
Authentication	STR testing.
Mycoplasma contamination	All cell lines tested negative for mycoplasma contamination.
Commonly misidentified lines (See ICLAC register)	None commonly misidentified cell lines were used.

Animals and other research organisms

Policy information about studies involving animals; ARRIVE guidelines recommended for reporting animal research, and Sex and Gender in Research

Laboratory animals	Bach1fl/fl mice and Bach1TG mice (male, 8 weeks,); db/db mice (male, 4weeks); ob/ob mice (male, 8 weeks).,C57BL/6J mice (male, 8 weeks); Mice were housed in standard cages with an SPF environment with a 12-hour light/dark cycle at a room temperature of 22°C ±2°C, humidity of 50%±5%, with free access to food and water.
Wild animals	The study did not involve wild animals.
Reporting on sex	There is limitation in this work. The mouse experiments were performed only in male's animals, which is indicated in the abstract and the discussion. For studies involving human research participants, the gender was considered in the study design and gender of participants was determined based on assigned.<br>At partial justification, male subjects are more likely to develop abnormal liver fat accumulation, non-alcoholic fatty liver disease, liver fibrosis, and liver tumors compared with female subjects, before the menopause. Therefore, we started with testing male mice in our study.
Field-collected samples	The study did not involve field-collected samples.
Ethics oversight	All animal studies were performed according to protocols approved by the Animal Ethics Committee of the Fudan University School of Basic Medical Sciences.

Note that full information on the approval of the study protocol must also be provided in the manuscript.
